# Traditional Uses of Animals in the Himalayan Region of Azad Jammu and Kashmir

**DOI:** 10.3389/fphar.2022.807831

**Published:** 2022-06-29

**Authors:** Maryam Faiz, Muhammad Altaf, Muhammad Umair, Khalid S. Almarry, Yahya B. Elbadawi, Arshad Mehmood Abbasi

**Affiliations:** ^1^ Department of Zoology, Women University of Azad Jammu and Kashmir, Bagh, Pakistan; ^2^ Department of Forestry, Range and Wildlife Management, The Islamia University of Bahawalpur-Pakistan, Bahawalpur, Pakistan; ^3^ College of Chemistry and Life Sciences, Zhejiang Normal University, Jinhua, China; ^4^ Department of Botany and Microbiology, College of Science, King Saud University, Riyadh, Saudi Arabia; ^5^ Department of Environment Sciences, COMSATS University Islamabad, Abbottabad, Pakistan; ^6^ University of Gastronomic Sciences, Pollenzo, Italy

**Keywords:** medicinal animals, zootherapy, ethnobiology, Kashmir, Himalayas

## Abstract

**Background:** The use of animals and animal-derived products in ethnopharmacological applications is an ancient human practice that continues in many regions today. The local people of the Himalayan region harbor rich traditional knowledge used to treat a variety of human ailments. The present study was intended with the aim of examining animal-based traditional medicine utilized by the population of the Himalayan region of Azad Jammu and Kashmir.

**Methods:** Data were collected from 2017 to 2019 through individual and group interviews. Data on traditional uses of animal products were analyzed, utilizing following indices such as the frequency of citation, use value, relative importance, similarity index, principal component analysis, and cluster analysis to find the highly preferred species in the area.

**Results:** Ethnomedicinal uses of 62 species of vertebrates and invertebrates were documented. Flesh, fat, bone, whole body, milk, skin, egg, head, feathers, bile, blood, and honey were all used in these applications. The uses of 25 animals are reported here for the first time from the study area (mainly insects and birds, including iconic species like the kalij pheasant, *Lophura leucomelanos*; Himalayan monal, *L. impejanus*; and western tragopon, *Tragopan melanocephalus*). The diversity and range of animal-based medicines utilized in these communities are indications of their strong connections with local ecosystems.

**Conclusion:** Our results provide baseline data valuable for the conservation of vertebrate and invertebrate diversity in the region of Himalayan of Azad Jammu and Kashmir. It is possible that screening this fauna for medicinally active chemicals could contribute to the development of new animal-based drugs.

## Introduction

Zootherapy is described as the use of animals or animal-derived materials to treat human ailments (Costa-Neto, 2005; Holennavar, 2015; Ahmad et al., 2021). The use of animals with medicinal properties continues to be a common practice worldwide. Zootherapy techniques and materials are utilized in traditional and nanomedicine for the treatment of different diseases (Kassam, 2002; Lawal and Banjo, 2007; Prakash and Verma, 2021). It is documented that almost 13% of the drugs used by traditional Chinese medicine are derived from vertebrates and invertebrates (Still, 2003). In Ayurvedic medicine, 15–20% of drugs contain vertebrate and invertebrate products (Unnikrishnan, 1998). In Tibetan medicine, the products of vertebrates and invertebrates are utilized in more than 111 drugs (Singh, 2000).

Many societies are rapidly losing their ethnopharmacological knowledge; so, documenting this knowledge before it is lost is increasingly important (Alves and Rosa, 2007; Löki et al., 2021; Mandal and Rahaman, 2022). Likely because of the dominance of plants in traditional medicine systems, the use of animals and animal-derived products in traditional medicine has been under-documented. Pakistan has a rich faunal diversity, including 195 species of mammals (Roberts, 1997), 668 species of birds (Mirza and Wasiq, 2007), 195 species of reptiles (Khan, 2006), and 24 species of amphibian studied by Khan (2010). To date, a number of studies have documented the use of animal parts in traditional medicine in different parts of Pakistan (Muhammad et al., 2018; Adil and Tariq, 2020; Aslam and Faiz, 2020; Mughal et al., 2020; Noor and Haider, 2020; Altaf et al., 2021; Haidar and Bashir, 2021; Ijaz and Faiz, 2021; Ijaz and Iftikhar, 2021; Saleem et al., 2021); however, ethnomedicinal uses of animals in Azad Jammu and Kashmir have never been reported.

Animals and its derived products are important elements in many traditional treatments (Ferreira et al., 2010; Albuquerque et al., 2012; Altaf and Faiz, 2021), and they have presumably utilized since prehistoric times (Alves et al., 2010; Prakash and Prakash, 2021). Traditional information can lead scientists to promising natural sources of new medicines, making it a powerful ally in the discovery of new drugs (Saleem et al., 2021; Habib, 2022). A suitable model for replicating contact dermatitis is phenol-induced ear edema. When phenol comes into direct contact with the skin, “keratinocytes” release chemical mediators that are crucial in prime contact irritation reactions, including as pro-inflammatory cytokines (Lim et al., 2004). These pro-inflammatory cytokines are made in a different way than those synthesized by PKC (as occurring in inflammations induced by croton oil). The rupturing of the “keratinocyte plasma membranes”, which leads to the liberation of pre-formed IL-1, as well as other inflammation mediators, is thought to be the cause of cutaneous irritations (Murray et al., 2007).

Zoonotic diseases are transferable diseases caused by infectious agents (such as viruses, bacteria, prions, or parasites) that can be transferred from a non-human animal to a human. Zoonotic diseases have caused a series of major global public health issues (malaria, yellow fever, avian flu, swine flu, West Nile virus, MERS, SARS, etc.), culminating in the current coronavirus health crisis (Altaf, 2016; Altaf, 2020). Different pathogens have different modes of transmission (Kruse et al., 2004; Van Vliet et al., 2017), so the risk of zoonotic diseases depends on the type of animals with which humans are in contact (as well as the duration and nature of contact) (Bilal et al., 2021). For example, the prevalence of diseases from fish to humans is very low (EHS, 2016b), while the risk of transmission from amphibians is higher due to human sensitivity to their porous skin (EHS, 2016a). The main aim of this study is to determine what animals local populations in Pakistan are in contact with in order to contribute to an understanding of the risk of zoonotic disease transmission due to ethnopharmacological uses of animals.

Human impacts on natural systems are complex. Many indigenous cultures have traditionally promoted ways of life that are relatively balanced in relation to the sustainability of their resource use. On the other hand, the forces of capitalism coupled with a conceptual nature–culture divide and propagated through the global spread of colonialism have resulted in extractive approaches to resource use that threaten the resilience of the majority of ecosystems. Ethnozoological research is critical to understanding the sustainability of biocultural systems (Fopa et al., 2020). Cultural uses of animal species (i.e., food, hunting, medicine, entertainment, religious practice, and trade) may promote beliefs and behaviors that help to conserve these animal species; however, if they are practiced unsustainably, or affected by commercialization or other political and economic factors, they may negatively affect or even endanger these animals. The use of animal species for traditional medicine and cultural purposes by local communities must also be considered in relation to other factors, such as changes in climate and habitat (Alves, 2012; Alves et al., 2018). There exists a global need to find new approaches to dealing with the present crisis of biodiversity loss (Boivin et al., 2016), and ethnozoology provides critical insights into the practices of local communities, allowing conservation efforts to effectively partner with resource stewards to promote the overall integrity of biocultural systems (Saunders, 2003; Dickman, 2010). This study on the medicinal uses of fauna by the rural and urban people of the Himalayan region of Azad Jammu and Kashmir is part of a broader research project to document the uses of animals by local communities throughout Pakistan (Muhammad et al., 2017a; Muhammad et al., 2017b; Muhammad et al., 2017c; Altaf et al., 2018; Altaf, 2020).

## Methods

### Description of the Study Area

**FIGURE 1:**
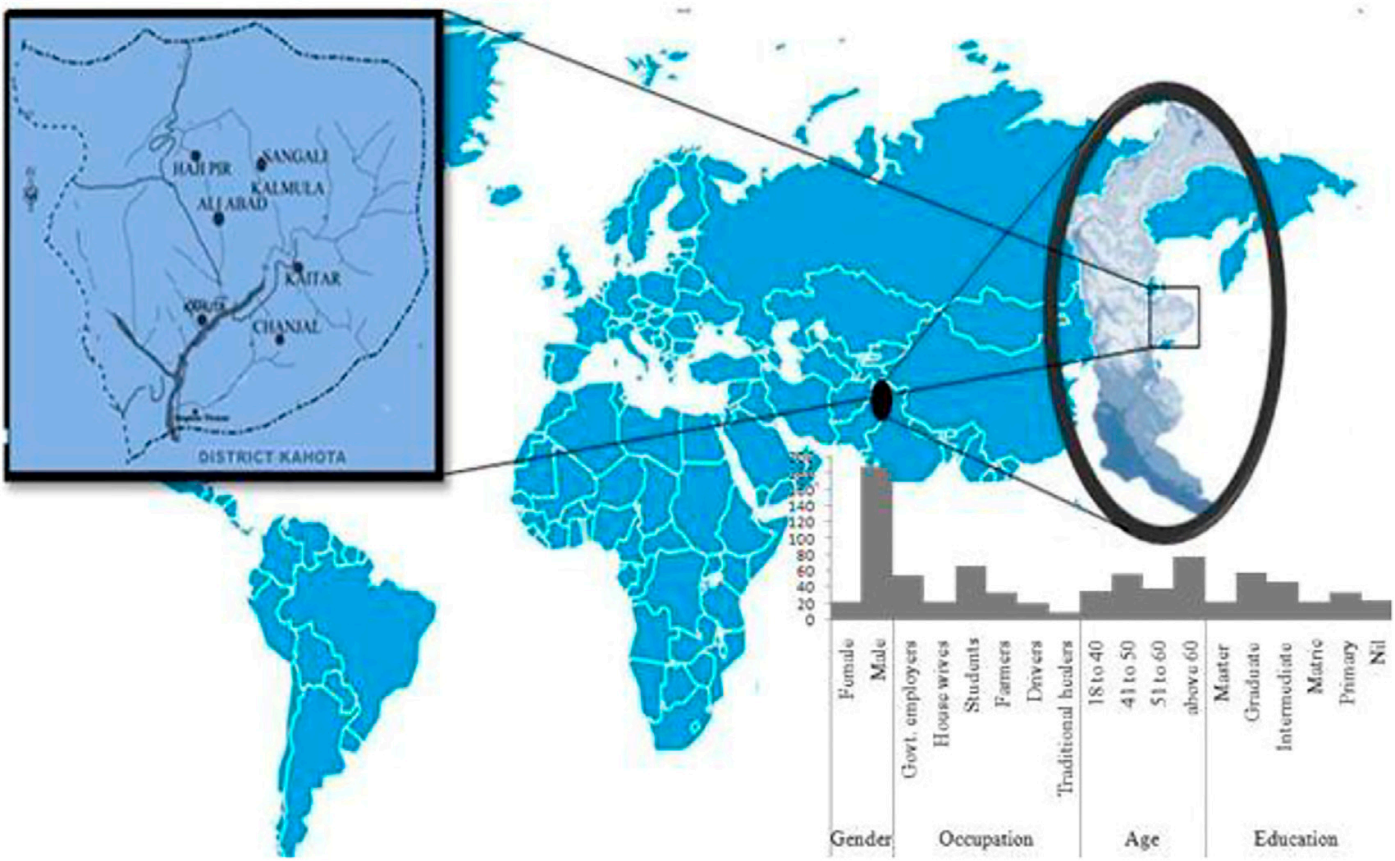
Map and respondent profile of the study area of the district Haveli.

Individual and group interviews were conducted in six different sites of the Himalayan region in Azad Jammu and Kashmir during 2017–2019 (Figure 1). The study area is located between 33° and 35° North latitude and 73° and 75° East longitude, in the foothills of Himalayas on the North East side of Pakistan, with an average elevation of 6,325 m in the north and 360 m in the south (Khan et al., 2017). Azad Jammu and Kashmir (AJ&K) is a cultural and geographical land of narrow, long, strip and occupies an area of 13,297 km^2^, with >4 million population. The main rivers of AJ&K are Jhelum, Poonch, and Neelum. The climate of this region is subtropical with an average rainfall of >150 cm. Spruce (*Abies pindrow*), Kail (*Pinus excelsa*), cheer (*Pinus willichiana*), deodar (*Cedrus deodara*), fur (*Pinus* spp.), and some other conifer species are dominant trees in AJ&K forests (PM, 2008; Ch et al., 2013; Khan et al., 2017; FWFD, 2021).

The study area is dominated by different tribal groups, such as Khawaja, Gujjar, and Rathor, which are the most common. Pahari, Kashmiri, and Gujjari are the major languages spoken, while Urdu is the official language, which is spoken as a second language by most people. The population of Haveli District was 112,000 in the census of 1998 and 157,000 in the census of 2015. The density was 262 people per sq. km in 2015. The average household size in the district is around 7, with most people living in joint family structures. The majority of the population lives in rural areas and is entirely Muslim. Most of the people (˃70%) in the study area are educated (Khan et al., 2017).

### Data Collection and Analysis

Before beginning fieldwork, consent was obtained from the “Department of Zoology, Women’s University of Azad Jammu and Kashmir, Bagh-Pakistan,” while questionnaires and interviews were arranged to record the ethnomedicinal uses of animals. Data were taken from respondents (*n* = 210) who included government employees, housewives, students, farmers, drivers, and customary wellbeing practitioners (Supplementary Table S1). Respondents were chosen based on their having basic awareness of folk medicines of wild animals. During the field survey, prior informed consent was obtained from each participant, and general standards/guidelines of the International Society of Ethnobiology (ISE) (http://www.ethnobiology.net/) and Consensus Statement on Ethnopharmacological Field Studies (ConSEFS) (https://www.journals.elsevier.com/journal-of-ethnopharmacology/) by Heinrich et al. (2018) were followed.

Field guides of mammals, birds, and herpetofauna *“Mammals of Pakistan”* (Roberts, 2005), *“Birds of Pakistan”* (Roberts, 1991, 1992), and *“Amphibian and Reptiles of Pakistan”* (Khan, 2006) were shown to informants to verify which species they described. Basic data on medicinal uses were then used to generate different indices including “frequency of citation,” “use value,” and “relative importance,” which were then analyzed using statistical methods including “similarity index,” “principal component analysis,” and “cluster analysis.”

### Quantitative Analysis

The ethnozoological data were analyzed by various indices, which include “FC” (frequency of citation), RI (relative importance), and UV (used-value).

### Frequency of Citation and Relative Importance

The frequency of citation is the number of respondents who described the medicinal uses of wild fauna species. The relative importance index was intended by the formula, as reported by Oliveira et al. (2010).

RI=PP+AC

where PP stands for pharmacological property quantity and AC is the maximum number of ailment categories treated by the most resourceful species divided by the number of ailment categories treated by a given species.

### Use Value and Similarity Index

The use value (UV) is the quantitative measure of the relative importance of specific animal species known locally. UV and the SI were calculated following the method reported previously (Trotter and Logan, 1986; Phillips and Gentry, 1993), using the formula:

UV=∑U/n

The number of citations per species is n, and the number of informants is U.

$$\text{SI}=\frac{{\text{S}}_{\text{a}}}{{\text{T}}_{\text{a}}}\left(0<\text{SI}<1\right)$$

Note: S_a_ = Similar documented ailment in the previous and present studies, T_a_ = Total documented ailment in the present study.

### Statistical Analysis

Data were analyzed in “Microsoft Excel 2010” (Microsoft, Redmond, WA, United States), whereas inferential statistical analysis was performed by using R software 3.6.3 and PAST 3.20 (Hammer et al., 2001). In addition, traditional uses of the body part(s) of animal species and their mode of application were represented in chord diagrams generated with the “circlize package (24)” in R software 3.6.1 (Gu et al., 2014).

## Results

**FIGURE 2:**
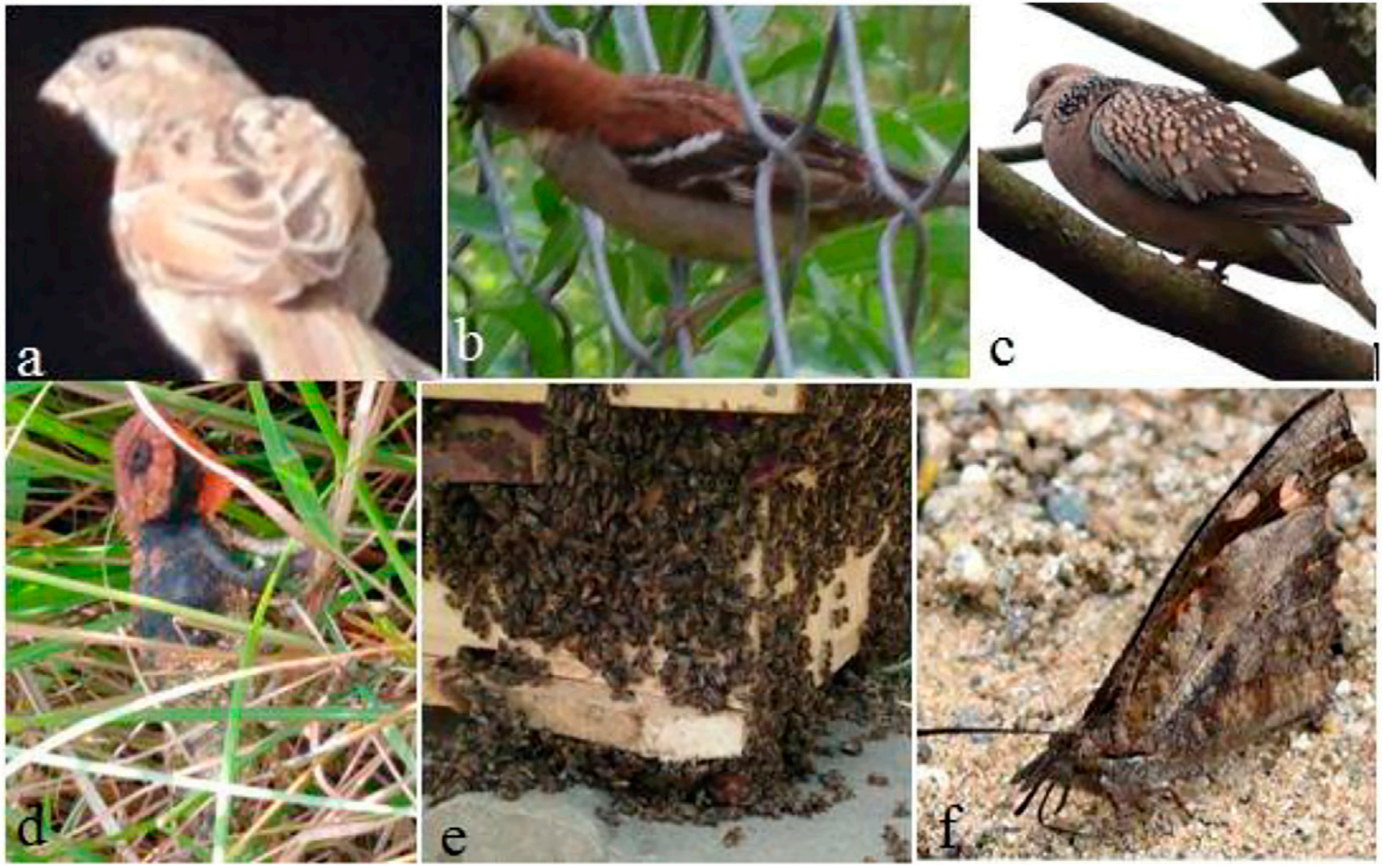
Statistically significantly important pictures (**(A)** house sparrow; **(B)** Russet sparrow; **(C)** spotted dove; **(D)** oriental garden lizard; **(E)** honey bee; **(F)** common beak) from study areas.

Data were gathered from 188 males and 22 females in the Haveli District. Majority of the informants were males, because due to cultural restrictions, usually females avoid conversation with strangers. Most of the data were collected from the rural area as majority of the inhabitants (*n* = 127) live there. Informants were common people, government employees, teachers, students, farmers, and shopkeepers. Among these, ˃90% were literate and the rest were illiterate. Most of the informants (*n* = 78) were of age more than 60 years, while young respondents (18–40 years) were 36 in number (Figure 1). Cow*, Bos taurus;* hen*, Gallus gallusd omesticus;* buffalo*, Bubalus bubalis;* duck, *Anas platyrhynchos domesticus;* hill pigeon, *Columba rupestris;* common pigeon*, Columba livia*; russet sparrow*, Passer cinnamomeus*; house sparrow*, Passer domesticus*; common hoopoe, *Upupa epops*; spotted dove*, Spilopelia chinensis*; oriental turtle dove, *Streptopelia orientalis*; sheep*, Ovis aries*; Himalayan monal*, Lophophorus impejanus*; camel, *Camelus dromedaries*; honey bee*, Apis mellifera*; chukar partridge*, Alectoris chukar*; alpine musk deer*, Moschus chrysogaster* and goat, *Capra aegagrus hircus* were the commonly utilized species in the region of Himalayan, AJ&K (Supplementary Table S1; Figure 2).

**FIGURE 3:**
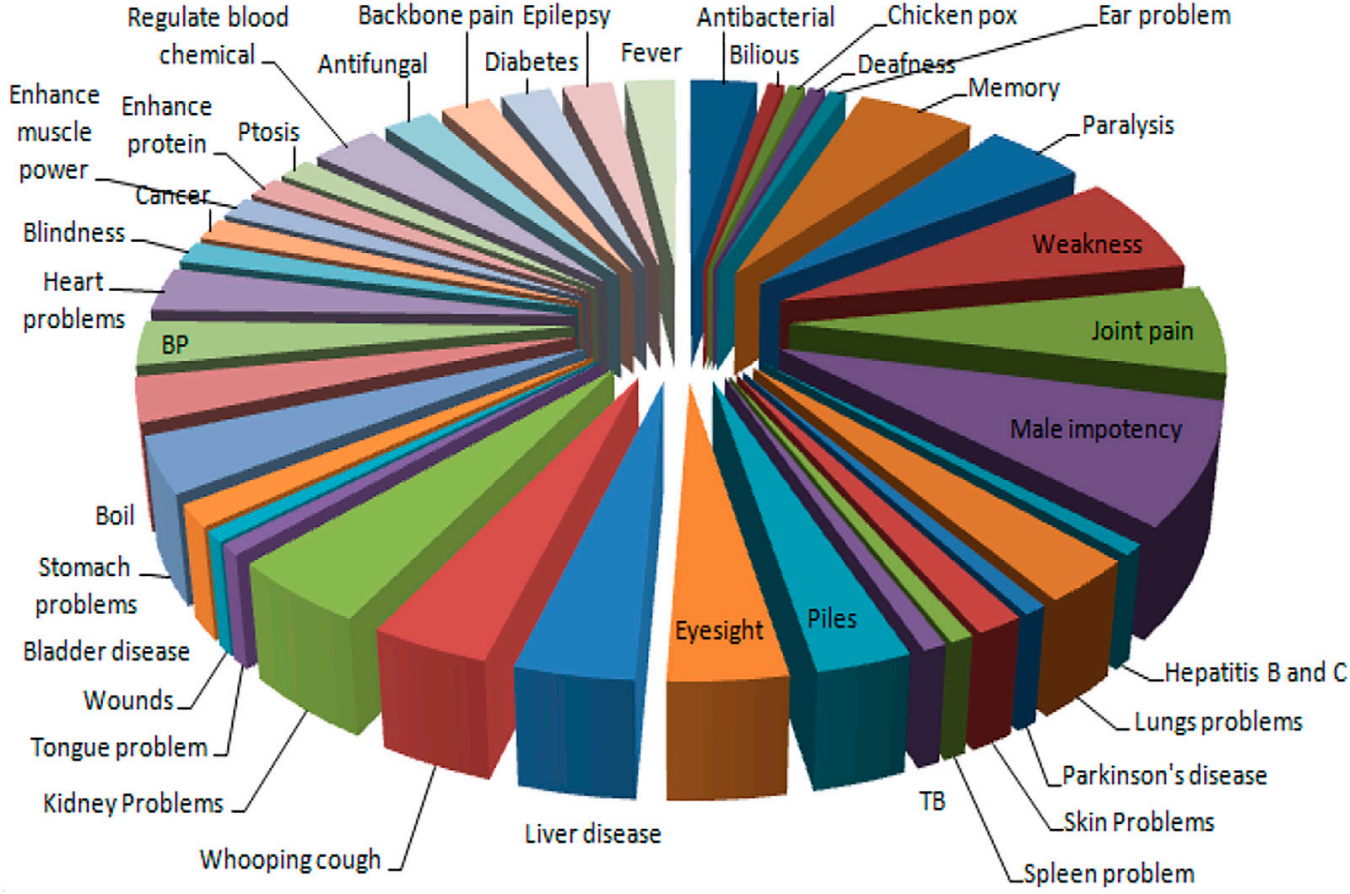
Comparative analysis of species used to cure various ailments in the district Haveli, Azad Jammu and Kashmir.

**FIGURE 4:**
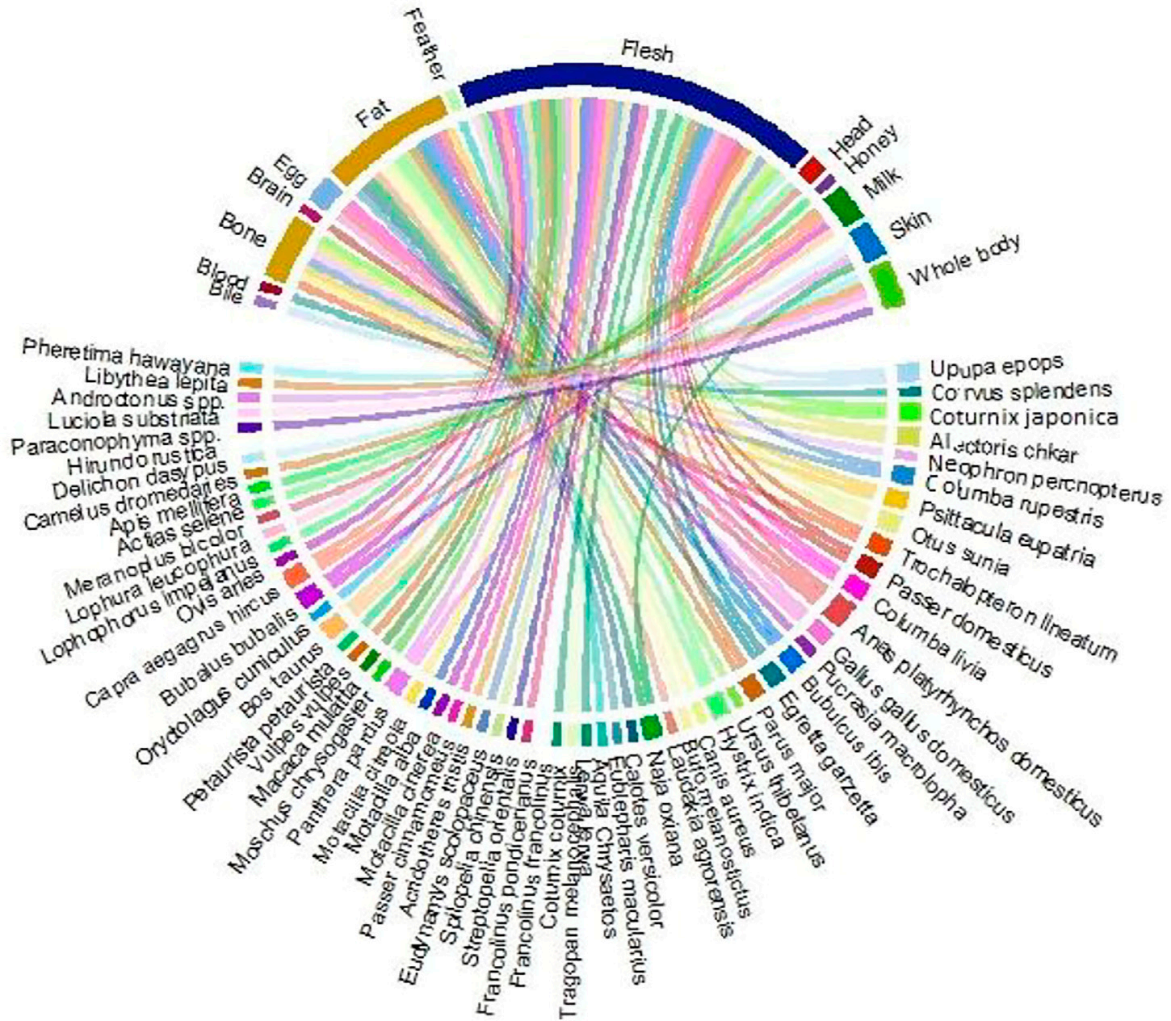
Body parts of animal species used in different recipes.

**FIGURE 5:**
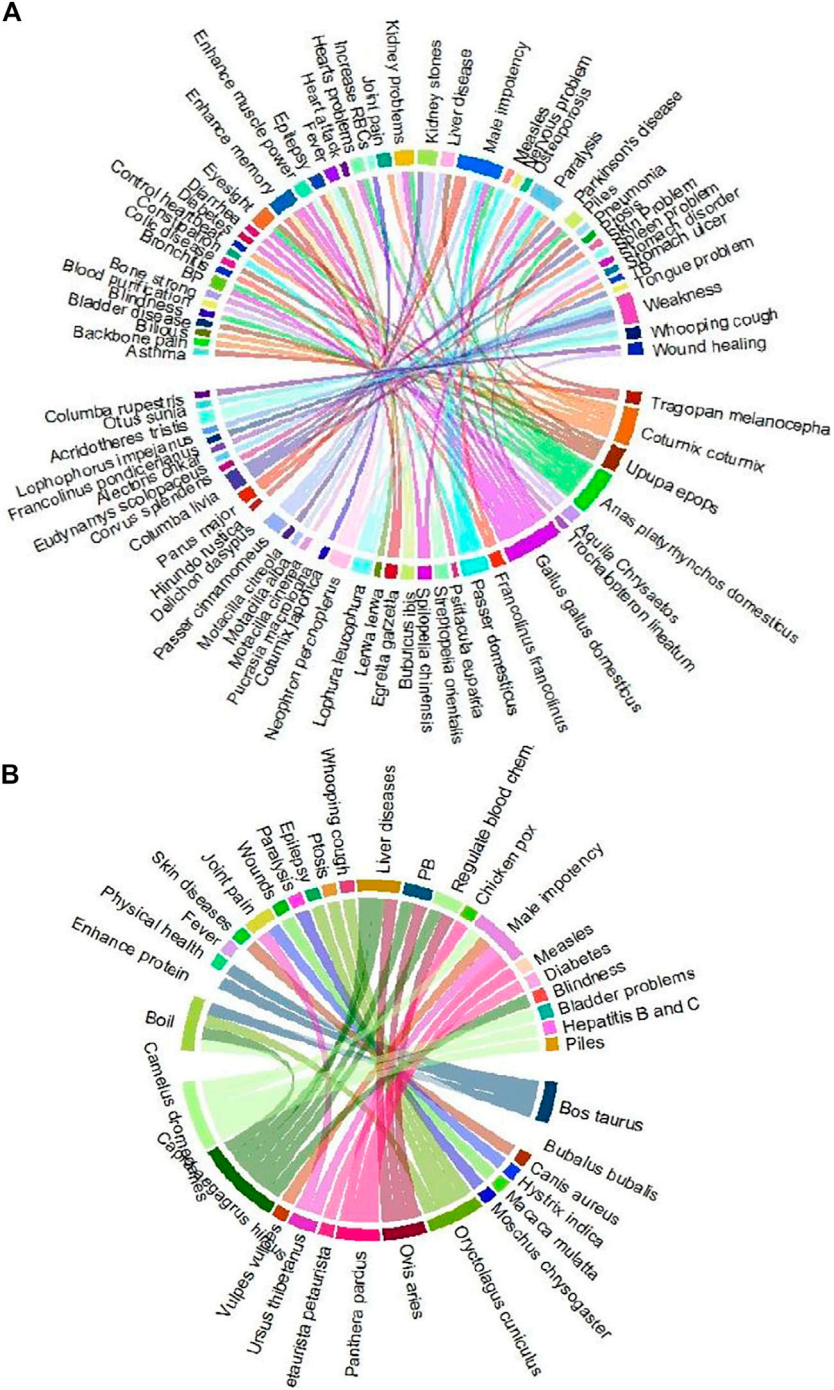
Animal species distribution according to the treatment of various ailments in the Himalayan region of Azad Jammu and Kashmir. Animal species are classified into different groups such as **(A)** birds and **(B)** mammals.

**FIGURE 6:**
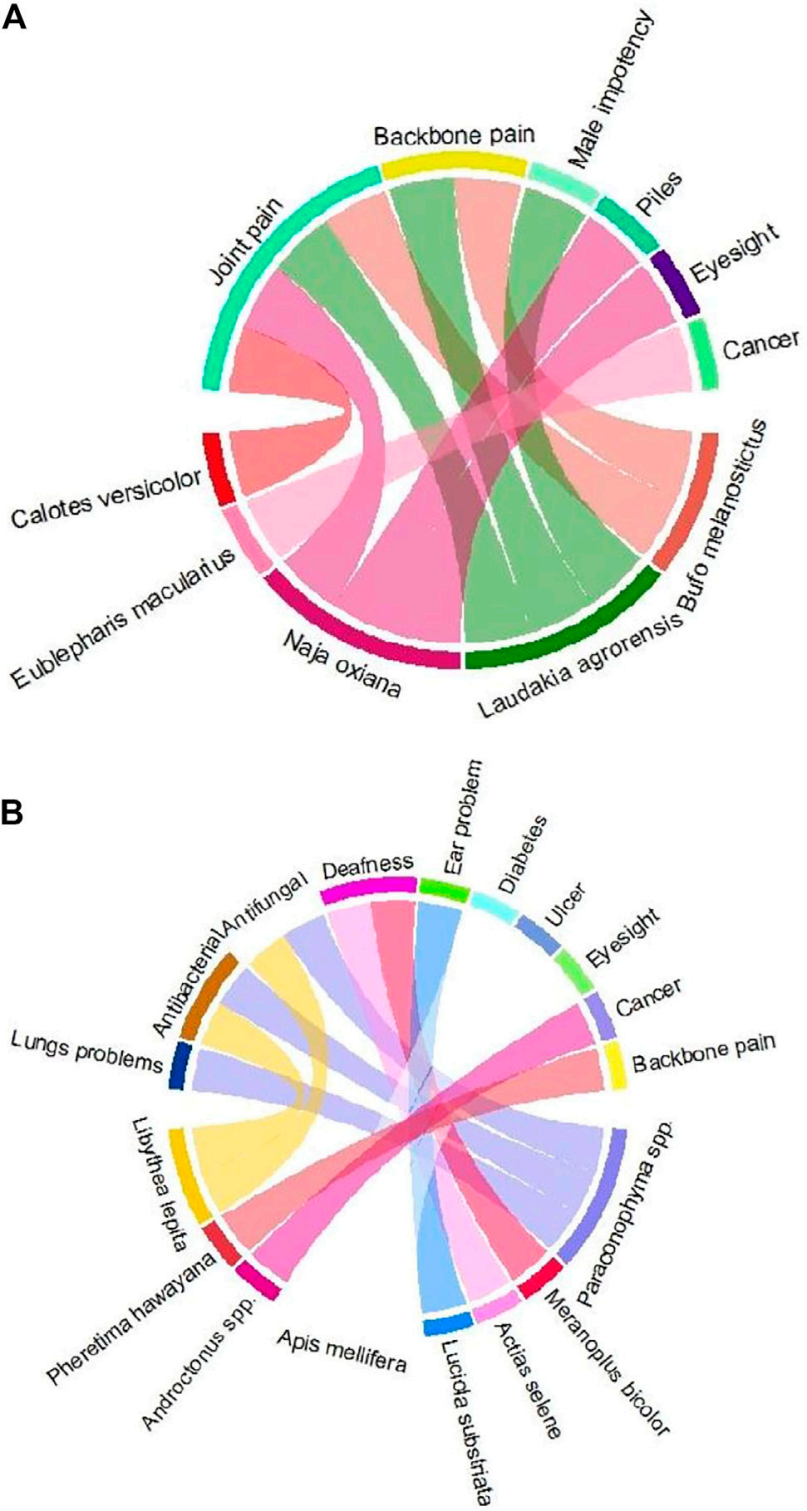
Animal species distribution according to the treatment of various ailments in the Himalayan region of Azad Jammu and Kashmir. Animal species are classified into different groups such as **(A)** reptiles and **(B)** invertebrates.

Thirty-nine diseases were treated with different animal parts and products (Figure 3), such as flesh, bone, whole body, milk, skin, egg, head, feather, bile, blood, and honey. Flesh was the most consumed (*n* = 35) body part, followed by bone, whole body, milk, skin, egg, head, feather, bile, blood, and honey (Figure 4). Local inhabitants use the fat of different species such as little egret (*Egretta garzetta*) and cattle egret (*Bubulcus ibis*) to treat memory and epilepsy, golden eagle (*Aquila chrysaetos*) to treat wound healing, and regulate blood chemical, Alexandrine parakeet (*Psittacula eupatria*) to treat memory, great tit (*Parus major*) to treat male impotency and skin problem, duck (*Anas platyrhynchos domesticus*) to treat kidney problems, heart problems, BP, male impotency, piles, blindness, and eyesight, Asiatic black bear (*Ursus thibetanus*) to treat joint pain and male impotency, Indian crested porcupine (*Hystrix indica*) to treat joint pain, Asiatic jackal (*Canis aureus*) to treat skin problems, Hazara gauk (*Duttaphrynus melanostictus*) to treat antibacterial and antifungal, agror agama (*Laudakia agrorensis*) to treat joint pain, backbone pain, and male impotency, brown cobra (*Naja oxiana*) to treat joint pain, piles, and eyesight, oriental garden lizard (*Calotes versicolor*) to treat joint pain, and leopard gecko (*Eublepharis macularius*) to treat cancer (Figures 5, 6).

Likewise, the meat of snow partridge, *Lerwa lerwa,* was used to treat fever; western tragopan*, Tragopan melanocephalus,* was used to treat lung problems and weakness; common quail*, Coturnix coturnix,* was used to treat bilious, heart problems, TB, joint pain, backbone pain, and paralysis; rain quail*, Coturnix japonica,* was used to treat and regulate blood chemical; chukar partridge, *Alectoris Chukar,* was used to treat weakness; black francolin*, Francolinus francolinus,* was used to treat joint pain and lung problems; grey francolin, *Francolinus pondicerianus,* and Himalayan monal, *Lophophorus impejanus,* were used to treat weakness; kalij pheasant, *Lophura leucomelanos,* was used to treat weakness, fever, and memory; common pigeon*, Columba livia,* was used to treat Parkinson’s disease, ptosis, and tongue problem; hill pigeon, *Columba rupestris,* was used to treat wound healing; spotted dove, *Spilopelia chinensis,* and oriental turtle dove, *Streptopelia orientalis,* were used to treat paralysis and enhance muscle power; Asian koel, *Eudynamys scolopaceus,* was used to treat spleen problem; oriental scopus owl, *Otus sunia,* was used to treat whooping cough; common hoopoe, *Upupa epops,* was used to treat stomach problems, liver disease, bladder disease, and eyesight; Asian house martin, *Delichon dasypus,* was used to treat male impotency; barn swallow, *Hirundo rustica,* was used to treat male impotency; streaked laughing thrush, *Trochalopteron lineatum,* was used to treat weakness; common myna, *Acridotheres tristis,* was used to treat whooping cough; russet sparrow, *Passer cinnamomeus,* was used to treat paralysis, male impotency, and liver; grey wagtail, *Motacilla cinerea,* white wagtail, *Motacilla alba,* and citrine wagtail, *Motacilla citreola,* were used to treat kidney problems; duck, *Anas platyrhynchos domesticus,* was used to treat kidney problems, heart problems, BP, male impotency, piles, blindness, and eyesight*;* hen, *Gallus gallus domesticus,* was used to treat kidney problems, heart problems, weakness, memory, eyesight, male impotency, diabetes, stomach problems, and BP; alpine musk deer, *Moschus chrysogaster,* was used to treat paralysis*;* Rhesus Macaque, *Macaca mulatta,* was used to treat wounds*;* red fox, *Vulpes vulpes,* was used to treat male impotency; giant red Himalayan squirrel, *Petaurista petaurista,* was used to treat diabetes*;* cow*, Bos taurus,* was used to treat and enhance protein, weakness, and boil; and *Bubalus bubalis* was used to treat fever and enhance protein (Figures 5, 6).

Similarly, bones of *Coturnix japonica*, *Alectoris chukar, Neophron percnopterus, Columba rupestris, Otus sunia, Trochalopteron lineatum,* and *Psittacula eupatria* were used to treat and regulate blood chemicals, weakness, stomach problems, kidney problems, heart problems, wound healing, whooping cough, and memory. Similarly, bones of *Paraconophyma* spp.*, Luciola substriata, Androctonus* spp.*, Libythea lepita,* and *Pheretima hawayana* were used to treat lung problems, antibacterial, antifungal, deafness, ear problems, diabetes, stomach problems, and eyesight. Further, the head of *Meranoplus bicolor* and *Actias selene* was used to cure deafness and antibacterial. Milk, feather, bile, blood, and honey were used to treat piles, diabetes, stomach problems, eyesight, male impotency, paralysis, measles, stomach problems, male impotency, and liver disease (Figures 5, 6).

In the same way, eggs of *Columba livia*, *Anas platyrhynchos domesticus,* and *Gallus gallus domesticus* were used to treat Parkinson’s disease, ptosis, kidney problems, stomach problems, heart problems, BP, male impotency, piles, blindness, eyesight, diabetes, wound healing, and memory (Figures 5, 6).

### Frequency of Citation

**FIGURE 7:**
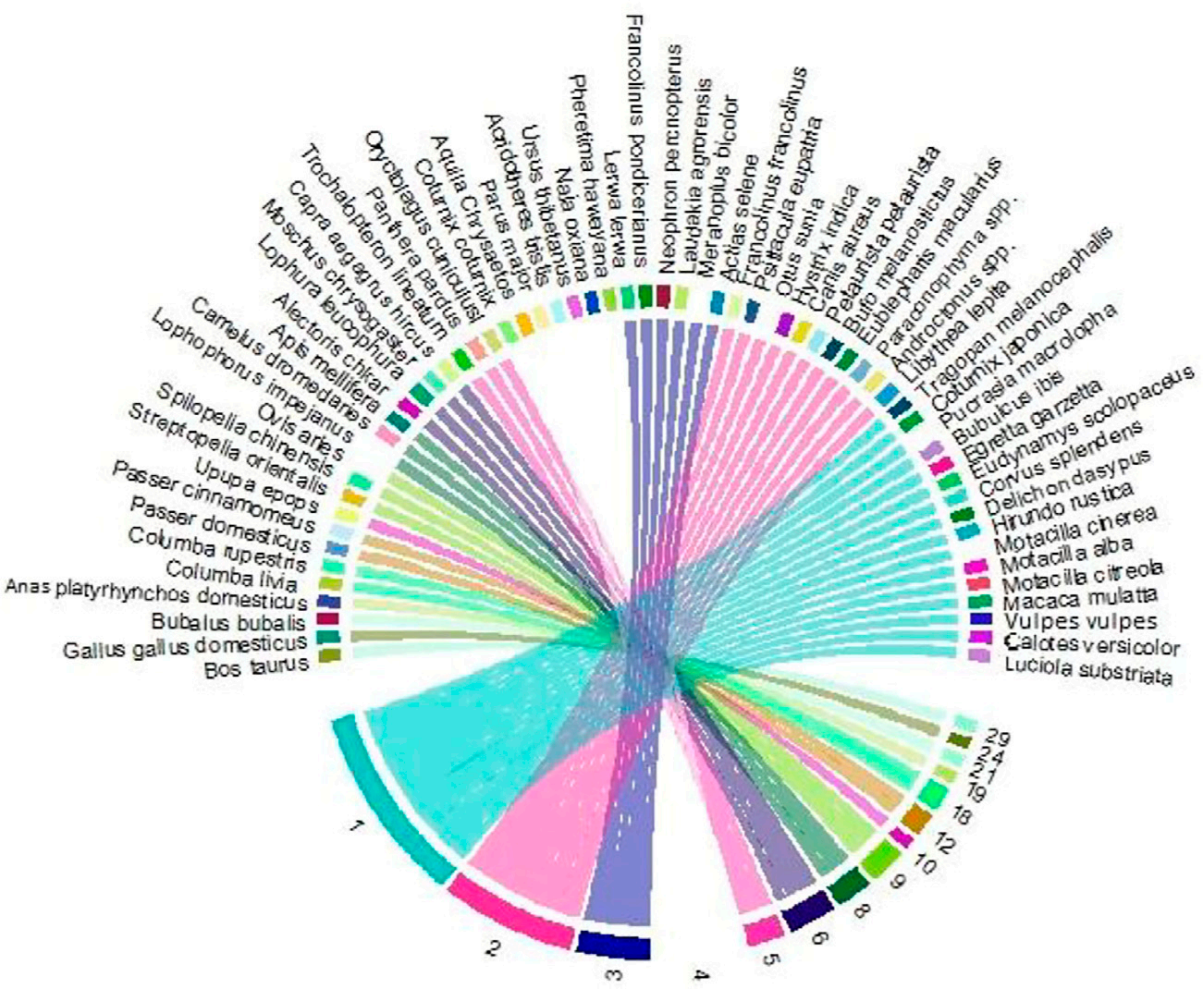
The relationship between the FC and the number of species application in Haveli districts.

Species of vertebrates and invertebrates documented by the most respondents have high “frequency of citation” scores, which ranged from 1 to 29 (Figure 7). COEP and COPH were documented as the most often consumed, with FC = 29 in the region of Himalayan, AJ&K. WTA, CQHD, CQTB, RQIR, KPJP, CEMM, CEEL, LEMM, LEEL, WVHA, GEBP, AKSP, HCPL, AMMP, BSMP, HSCP, GWKS, WWKS, CWKS, DUES, RMW, RFMI, GLJP, and FFEP had the lowest frequencies of citation (*n* = 1) (codes are written in Supplementary Table S1).

### Relative Importance

The “relative importance” values are presented in Supplementary Table S1. Most animals were documented to be highly versatile in their utilization (RI = 3.45) such as CQB, CQJP, CQBP, CQPL, CPPD, HPWH, HSMI, HSPL, RSPL, RSMI, DUKP, DUHP, HNKP, HNMI, PPCP, PPMI, COEP, and COPH (Figure 7), while the lowest value of RI (0.18) was recorded for DUES.

### Use Value

Among the reported wild animal species, the highest “UVs” (maximum of 1.0) were for HSPL, RSPL, RSMI, DUKP, DUHP, HNKP, HNMI, PPCP, PPMI, COEP, COPH, CQB, CQJP, CQBP, CQPL, CPPD, HPWH, and HSMI (codes are presented in Supplementary Table S1). The lowest UVs of 0.05 were noted for DUES. The high “UVs” of these species showed their widespread use in the healing of ailments.

### Similarity Index

Out of the total, 49 species have zero similarity index; this shows that the present study has a lot of novel data. The similarity index of *Gallus gallus domesticus* is 0.067 followed by *Capra aegagrus hircus* (SI = 0.056), *Camelus dromedaries* (0.17), *Passer domesticus* (0.2), *Laudakia agrorensis* (0.2), *Calotes versicolor* (0.2), *Passer cinnamomeus* (0.25), and *Naja oxiana* (0.34). *Columba rupestris, Eudynamys scolopaceus, Corvus splendens,* and *Acridotheres tristis,* which have a similarity index of 1 (Supplementary Table S1).

### Principal Component Analysis and Cluster Analysis

**FIGURE 8:**
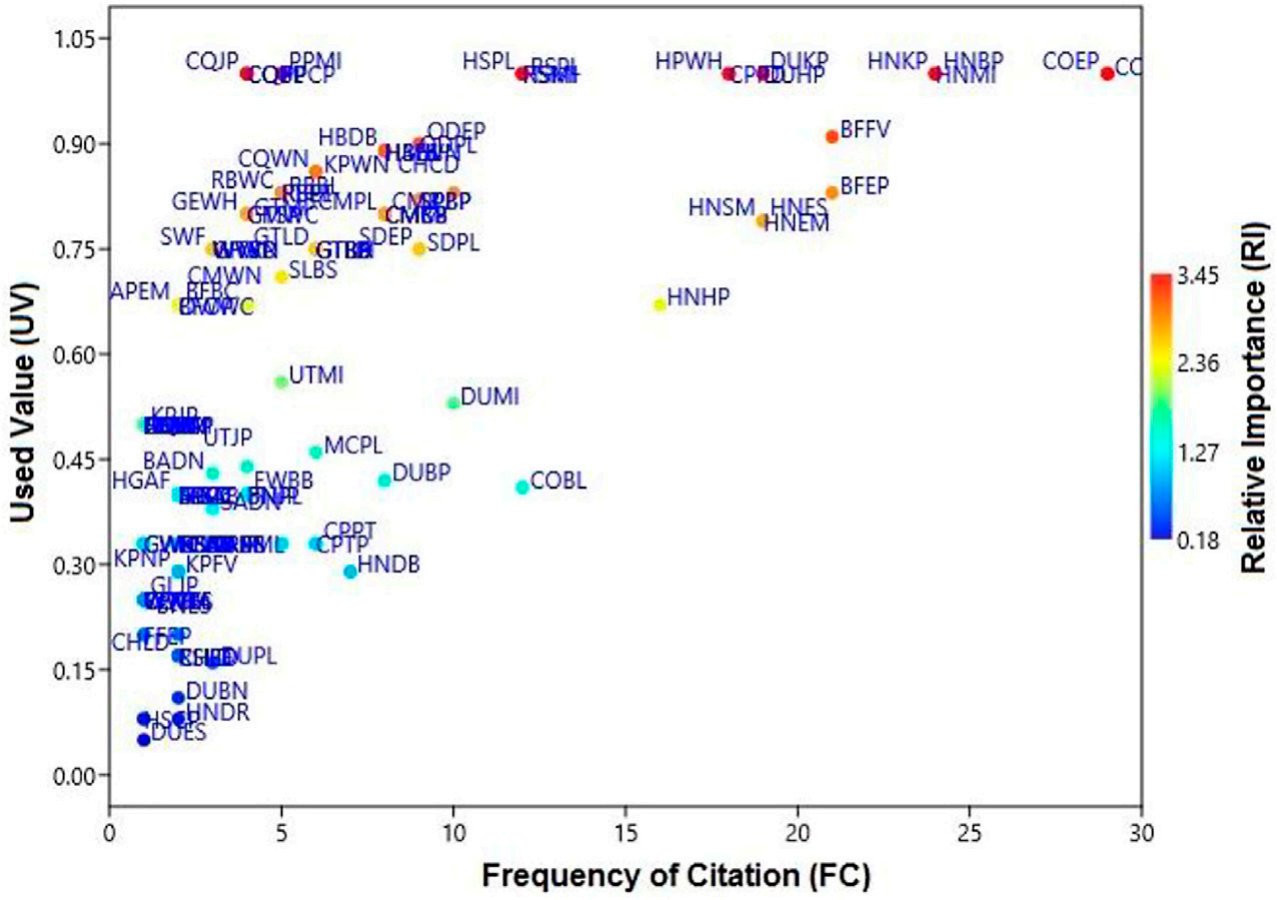
Relationship between the frequency of citation (FC), use value (UV), and relative importance (RI) for a particular disease (UV). Codes represent the species names of birds that appear in Supplementary Table S1.

**FIGURE 9:**
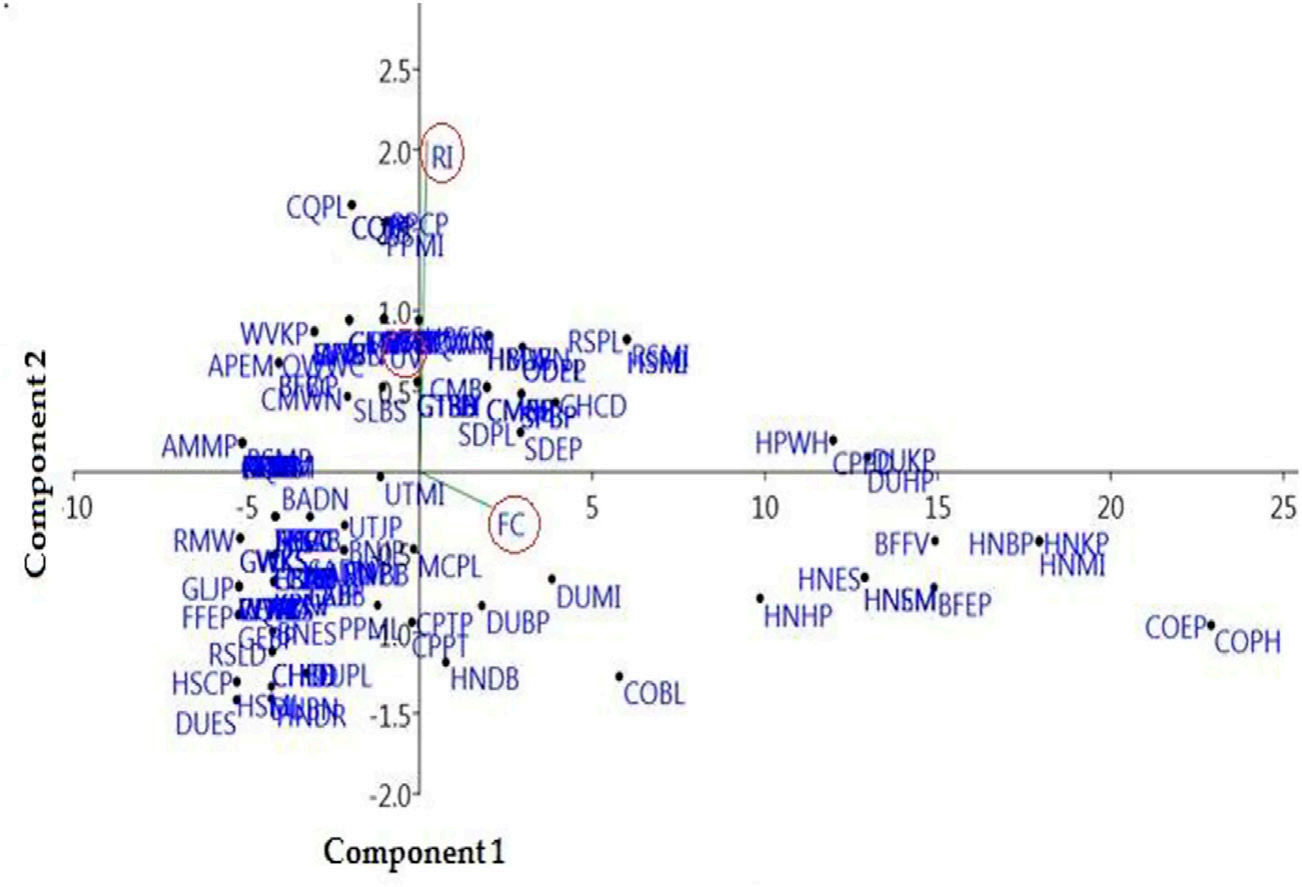
Principal component analysis (PCA) (codes are present in Supplementary Table S1), showing the positions of the arrows relative to components 1 and 2, shows how strongly independent variables (UV, FC, and RI) are correlated with each other from the district Haveli.

Statistical analysis with the assistance of “PCA” showed that the first two axes of the “PCA” has 100% variation and “PC 1” and “PC 2” have 98.5 and 1.5% variations, respectively (Figures 8, 9). Variables loaded onto the *x*-axis “PC 1” includes FC (*r* = 0.99454), UV (*r* = 0.029041), and RI (*r* = 0.10028), while “PC 2” included FC (*r* = -0.1044), UV (*r* = 0.27713), and RI (*r* = 0.95515). The “Cluster analysis” depicted various groups and sub-groups, which are distinguished on the basis of the source of the number of informants (Figures 8, 9). The results of PC1 exhibit positive correlations between FC, UV, and RI, while that of PC2 indicate a negative correlation with FC and a positive correlation with UV, as well as RI variables.

**FIGURE 10:**
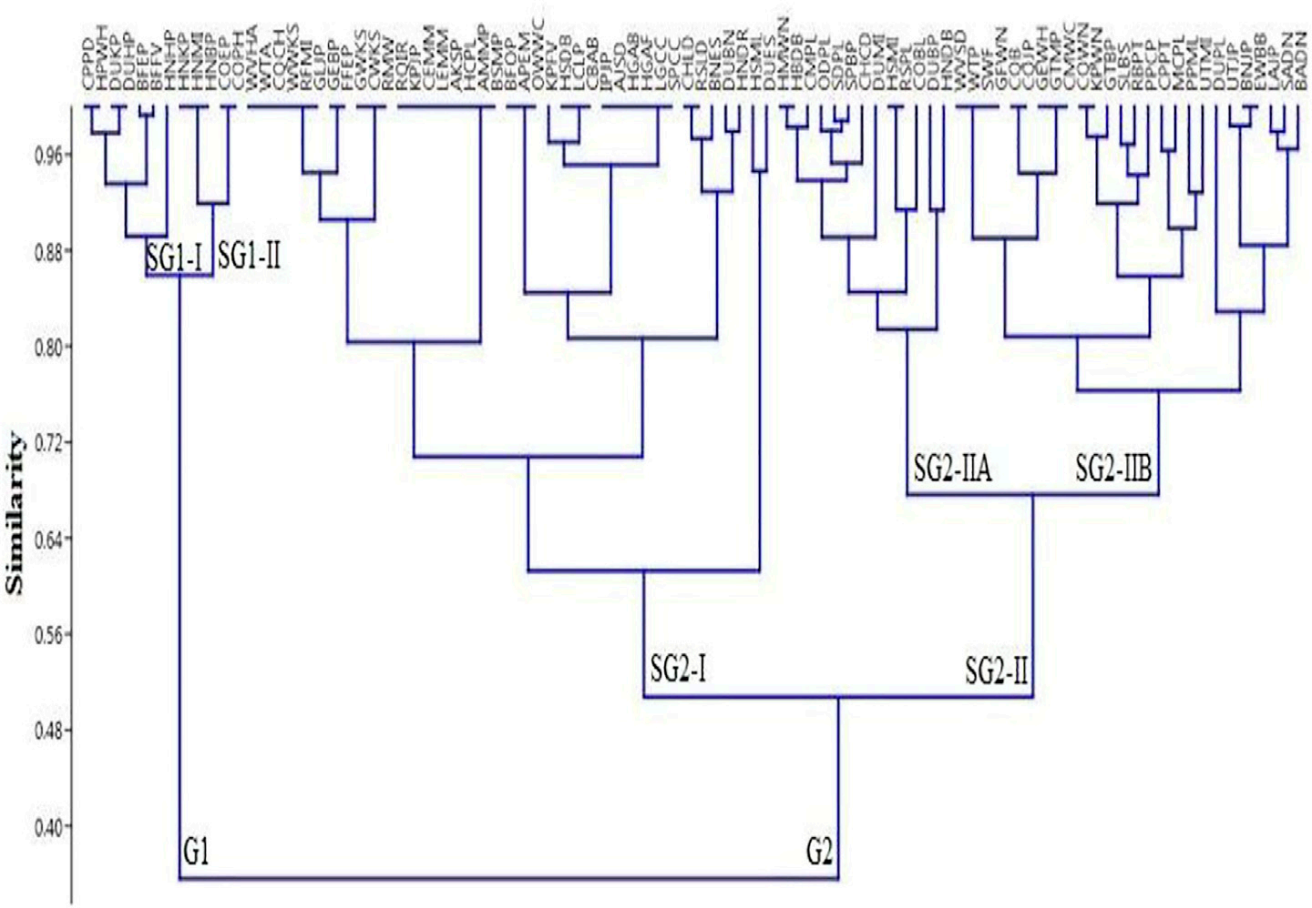
Cluster analysis showing the similarities among species (codes are present in Supplementary Table S1) in different variables (UV, FC, and RI) of the district Haveli.

The analysis demonstrated that two groups are noted in the “cluster analysis” in the Himalayan region, AJ&K, i.e., “group one” (G1) and “group two” (G2). “G1” and “G2” have a similarity of almost 0.8 points. G1 is further divided into two groups known as SG1-I (subgroup 1-I) and SG1-II (subgroup 1-II), while both have a similarity of approximately 0.5 points. Likewise, G2 is further divided into two groups known as SG2-I (subgroup 2-I) and SG2-II (subgroup 2-II), while both have a similarity of about 0.6 points. SG1-II has the following species of animals with diseases coded as COEP, COPH, HNBP, HNMI, HNKP, HNEM, and HNSM; while SG1-II has the following BFFV, BFEP, DUHP, DUKP, HPWH, and CPPD (Figure 10).

## Discussion

In ethnozoological research, socio-demographic data on respondents (age, gender, occupation, ethnicity, and education) are incredibly useful, as this component plays a key role in interpreting and analyzing the feedback received (Easthope, 1995; Hanif et al., 2019). Male respondents made up 94% contribution, whereas female respondents were rare in the present study. This is because most of the females are housewives and do not meet with strangers, so more males are selected for interviews. Altaf et al. (2017) discovered similar results in a research of ethnomedicinal and cultural activities of mammalian and avian in the region of Punjab, Pakistan. In fact, males hunted animals for food as well as for medicine, which could explain our findings. Additionally, the informants in village areas had more knowledge and information regarding the use of species for human ailments when compared to the informants in urban regions. These results were alike to earlier information from the district of Negev, Israel (Friedman et al., 1986).

The inhabitants of the region of Himalayan in AJ&K reported the ethnomedicinal uses of 62 animal species to treat 39 different diseases including male impotency, weakness, joint pain, memory loss, paralysis, piles, eyesight, stomachache, whooping cough, liver, and kidney problems among others in the present study. Similarly, 32 animal species, invertebrates and vertebrates, for treating 37 types of ailments were reported in southern regions of KPK, Pakistan. The major treated ailments were night blindness, epilepsy, cancer, hepatitis, asthma, paralysis, whooping cough, and brain hemorrhage (Mussarat et al., 2021). They reported the use of *Gallus gallus domesticus* for joint pain, blood pressure, weakness, hepatitis, diabetes, *Capra hircus* for hepatitis C, night blindness and joint pain, *Passer domesticus* for abdominal pain, and *Ovis aries* for the regulation of blood level, which supports our findings.

Birds were the most regularly used animal group for therapeutic purposes in our study. Previous findings revealed that wild birds are used as a food source in many parts of the world, including Pakistan (Arshad et al., 2014; Altaf et al., 2017; Mughal et al., 2020), India (Jaroli et al., 2010; Chinlampianga et al., 2013), Brazil (Alves et al., 2013; Teixeira et al., 2014), and Philippines (Ploeg and Weerd, 2010). Bird species are commonly used to treat various human ailments such as body pain, arthritis, respiratory disorders, gastric ulcers, obesity, and piles in the present study. Previous reports also showed that bird species are utilized in different folk therapies e.g., infertility, asthma, abscess, anemia, body weakness, body strength, bronchitis, breathing trouble, enhanced memory, immune enhancer, fever, flue, epilepsy, menorrhagia, paralysis, puberty in young girls, skin diseases, sexual power, and wound healing (Arshad et al., 2014; Vijayakumar et al., 2015a; Bagde and Jain, 2015; Vijayakumar et al., 2015b; Aloufi and Eid, 2016; Chattha et al., 2017; Hakeem et al., 2017; Ali et al., 2018; Mughal et al., 2020; Haidar and Bashir, 2021). In fact, parts or products of bird species are highly nutritious food and composed of “calcium,” “chlorine,” “iron,” “phosphorus,” “potassium,” “sodium,” “glycogen,” “lactic acid,” “lipids,” “magnesium,” “nitrogenous compounds,” “non-nitrogenous compounds,” and “water” (Keeton and Eddy, 2004; Hui, 2012; Cheung and Mehta, 2015). Moreover, birds are also connected with superstitious beliefs, such as people of the local area who believe that ducks and gooses are the sign of prosperity. However, in some reports from Pakistan, mammals are most used animals in ethnomedicines (Altaf et al., 2018; Mussarat et al., 2021).

It has been documented that omega-3 fatty acid in vertebrates’ fats decreases inflammation (Wilson, 2015; Ijaz and Faiz, 2021). Ethnobiologist documented that fats are used to treat a neurological disorder, atherosclerosis, thrombotic, and aging affects (Breteler, 2000; Haag, 2003).

Meat is composed of water, nitrogenous compounds, lipids, non-nitrogenous compounds, glycogen, lactic acid, sodium, magnesium, calcium, chlorine, potassium, iron, and phosphorus (Keeton and Eddy, 2004; Hui, 2012). Meat composition is different due to the impacts of different environmental and internal factors like feeding, muscle, animal species, breed sex, etc. (Cheung and Mehta, 2015). Beef, poultry, lamb, fish, and pork are the most common meats consumed in the world. Camel meat, on the other hand, is renowned in a few nations, particularly in dry and semi-arid regions, as the principal source of animal protein that equals, if not surpasses, the economical value of other meats (Williams, 2007; Schönfeldt and Gibson, 2008; Abrhaley and Leta, 2018; Haidar and Bashir, 2021).

It is documented that bones contain up to “95%” elastic protein, collagen fibers, as well as inorganic minerals such as calcium and phosphate. They improve bone fractures (Hall, 2011). Different species of animals, i.e., cinereous vulture, goat, alpine musk deer, crow, crab-eating macaque, common carp, fruit bat, deer, horse, and Indian gagata, were used for different ailments like improving wounds, digestion, heart strength, ear aches, lumbago, skin, chest pain, and urine problems (Ghosh et al., 2013; Vallejo and González, 2014; Vijayakumar et al., 2015a; Vijayakumar et al., 2015b; Yeshi et al., 2017; Altaf et al., 2018; Bullitta et al., 2018; Altaf, 2020; Abbasi, 2021; Ijaz and Iftikhar, 2021; Riaz and Altaf, 2021).

Eggs are an ideal source of protein and a balanced source of nutrients for humans of all ages, as well as also a supply of vitamins and other compounds and elements like “A,” “B6,” “B12,” “folic acid,” “phosphorus,” “selenium,” “amino acid,” and “iron.” Eggs are utilized to treat low blood pressure, fever, cold, weakness, breast cancer, weight loss, weak eye side, cold, bones, teeth, CNS, sprains, eye-each, BP, nourishing, bronchitis, asthma, burst furuncles, hemorrhoids, diabetes, jaundice, indigestion, to ease birth, diabetes, sinusitis, bronchitis, shortness of breath, rheumatism, stuffy nose, nervous problems, flu, weak bones, furuncle, burns, night blindness, weakness, sore throat, and otic infectivity (Padmanabhan and Sujana, 2008; Alves et al., 2010; Lohani, 2010; Oliveira et al., 2010; Alonso-Castro et al., 2011; Lohani, 2011b; Jacobo-Salcedoa et al., 2011; Alves et al., 2012; Barros et al., 2012; Haileselasie, 2012; Souto et al., 2012; Bagde and Jain, 2013; Betlu, 2013; Kim and Song, 2013; Martínez, 2013; Chellappandian et al., 2014; Bagde and Jain, 2015; Altaf et al., 2017; Dey et al., 2017; Altaf et al., 2018; Tariq, 2020). The egg is made up of structures that provide the optimum environment for the growth and development of an embryo. It is one of the biggest sources of essential nutrients for humans except the vitamin C. Eggs are surprisingly delicious and healthy foods used in different ways (Tariq, 2020).

Milk is one of the oldest foods (Wiley, 2015) and at the same time the most important one (Spreer, 1998). Milk of mammalian species consists of fats, proteins, lactose, ash, water, and solids (Guo et al., 2007; Hamad and Baiomy, 2010; Ballard and Morrow, 2013; Grădinaru et al., 2015; Merlin Junior et al., 2015; Getaneh et al., 2016; Kula and Tegegne, 2016; Abdullahi, 2019). Milk is utilized to cure a variety of sicknesses like hepatitis, measles, body pain, cancer, *tuberculosis*, diabetes, eye pain, whooping cough, cataract, sexual power, arthritis, and gastritis (Lev, 2003; WHO, 2005; Padmanabhan and Sujana, 2008; Alves et al., 2009; Benítez, 2011; Lohani, 2011b; Mishra et al., 2011; Yirga et al., 2011; Alves et al., 2012; Barros et al., 2012; Haileselasie, 2012; Martínez, 2013; Alonso-Castro, 2014; Betlloch Mas et al., 2014; Mootoosamy and Mahomoodally, 2014; Vijayakumar et al., 2015b; Borah and Prasad, 2017; Yeshi et al., 2017; Altaf et al., 2018; Aslam and Faiz, 2020).

Feathers are used because they are cheap and environmentally friendly for biomaterials. Feathers consist of α-helix and β-sheet. Bird feathers are utilized for decoration as well as for toys. Feathers of various species are used in traditional medicine, e.g., *Ceryle rudis, Nothura boraquira, Phalacrocorax brasilianus, Meleagris gallopayo, Coragyps atratus, Coryus splendens, Corythaeola cristata,* and *Columba livia,* which are utilized for the cure of cough, typhoid, headache, flu, asthma, alcoholism, love poison, and cough (Padmanabhan and Sujana, 2008; Alves et al., 2009; Lohani, 2011a; Jacobo-Salcedo et al., 2011; Haileselasie, 2012; Bezerra et al., 2013; Martínez, 2013; Alonso-Castro, 2014; Bobo et al., 2015; Vijayakumar et al., 2015b; Dos Santos Soares et al., 2018; Adil and Tariq, 2020). Feathers are utilized for various reasons, e.g., as a micro- and nanoparticle, bio-sorbent, enhance the viability of the cell, modify the antibacterial activity, and dressing of wounds, as well as in the cosmetic industries. Graphene oxide and derivatives are used as a biomaterial, films of thermoplastic, regenerated fibres, for ruminants as protein, feeding supplement, fire-resistant substance, handspun yarn, processing of leather, in the electrode material, formation of paper, textile fibers, bio-fertilizer, reformation of tissue, bio-composites, bio-plastic, and wound healing (Coward-Kelly et al., 2006; Karthikeyan et al., 2007; Reddy and Yang, 2007; Poole et al., 2009; Rouse and Van Dyke, 2010; Zhan and Wool, 2011; Gurav and Jadhav, 2013; Reddy et al., 2014a; Reddy et al., 2014b; Flores-Hernández et al., 2014; Manivasagan et al., 2014; Tsuda and Nomura, 2014; Xu et al., 2014; Amieva et al., 2015; Khajavi et al., 2016; Sharma et al., 2017a; Sharma et al., 2017b; Kumar et al., 2017; Tesfaye et al., 2017; Wang et al., 2017; Ramakrishnan et al., 2018; Nanthavanan et al., 2019; Adil and Tariq, 2020).

Honey is composed of “sugars” (Kamal and Klein, 2011), “disaccharides,” “water,” “proteins” (Moreira et al., 2007; Won et al., 2009; Sak-Bosnar and Sakač, 2012), “amino acids” (Hermosí;n et al., 2003; Iglesias et al., 2006), “vitamins” (Bonté and Desmoulière, 2013), “minerals” (Alqarni et al., 2014), “organic acids,” “phenolic compounds” (Andersen and Markham, 2005), and “solid particles” (Castro-Vázquez et al., 2007) as well as “volatile compounds” (Da Silva et al., 2016). Honey is used as a remedy in traditional medicine to cure gastritis, snake-bite, cold, myalgia, eye infection, teething in child, dark spots, skin, diarrhea, expectorant, migraine, allergy, burns, wounds in the stomach, spleen, toothache, mouth, influenza, hypertension, atherosclerosis, diabetes mellitus, Alzheimer’s disease, cancer, urinary system, throat pain, asthma, acidity obesity, cough, and tonsils (Mahawar and Jaroli, 2006; Padmanabhan and Sujana, 2008; Dixit et al., 2010; Jaroli et al., 2010; Oliveira et al., 2010; Abbasi et al., 2011; Benítez, 2011; Deb and Haque, 2011; Lohani, 2011b; Yirga et al., 2011; Barros et al., 2012; Erejuwa et al., 2012; Haileselasie, 2012; Chinlampianga et al., 2013; Betlloch Mas et al., 2017; Mootoosamy and Mahomoodally, 2014; Sreekeesoon and Mahomoodally, 2014; Vallejo and González, 2014; Vijayakumar et al., 2015b; Waykar and Alqadhi, 2016; Yeshi et al., 2017; Altaf et al., 2018; Altaf and Umair, 2020). Honey is also utilized in nano-medicine to cure various ailments and acts as anti-apoptosis, anti-proliferative (Oršolić, 2009; Li et al., 2010; Mandal and Mandal, 2011; Vallianou et al., 2014), anti-diabetic, antioxidant (Omotayo et al., 2010; Erejuwa, 2014; Bobiş et al., 2018), antibiotic, anti-cataract, anti-inflammatory, antifungal and endophthalmitis (Rhone and Basu, 2008; Vit and Jacob, 2008; Cernak et al., 2012; Salehi et al., 2014), blood pressure, heart problems (Al-Waili et al., 2013; Aluko et al., 2014), antibacterial, antioxidant (Francis et al., 2015; El-haskoury et al., 2018), and oxidative stress (Zhao et al., 2018).

COEP (flesh of *Bos taurus* enhances the amount of protein) and COPH (milk of *Bos taurus* is used to treat weakness) were documented as the most often consumed with FC = 29 in the Himalayan region of Azad Jammu and Kashmir. Most animals were versatile in the context of utilization (RI = 3.45), such as CQB (flesh of *Coturnix coturnix* is used to treat bilious), CQJP (flesh of *Coturnix coturnix* for joint pain), CQBP (i *Coturnix coturnix* is used to treat backbone pain), CQPL (flesh of *Coturnix Coturnix* is used against paralysis), and CPPD and HPWH (flesh of *Columba livia* and *C. rupestris* is used to treat Parkinson’s disease). The maximum relative importance is an indication of high accessibility and affordability of a species (Umair et al., 2019). Animal species with high RI values could be focused to evaluate their pharmacological and therapeutic potential. Therefore, statistical analysis is of significant value in ethnobiological studies because it facilitates the researcher in the selection of appropriate species and their body parts for chemical profiling and pharmacological/clinical studies. The ethnopharmacological data were calculated through “PCA,” which allocated for the ordination of designs in terms of three variables, i.e., FC, UV, and RI. Statistical analysis with the help of “PCA” showed that the first two axes have 100% difference and “PC 1” has 98.5% and “PC 2” has 1.5% variations. These findings were in agreement with other studies (Altaf, 2020).

### Novelty of Data

The folklore is an animal-based-medicinal concept of populations of the region of Himalayas, AJ&K. It means that people have a strong association with the ecosystem. For the first time, medicinal uses of animals from Azad Jammu and Kashmir were investigated. Furthermore, applications of 49 animal species are used to cure different diseases in humans. Out of 35 avian species, 28 species (i.e**.,** *Lerwa lerwa, Tragopan melanocephalus, Coturnix Coturnix*, *Coturnix japonica, Alectoris Chukar, Francolinus francolinus, Francolinu spondicerianus, Pucrasia macrolopha, Lophophorus impejanus*, *Lophura leucomelanos, Bubulcus ibis*, *Egretta garzetta*, *Neophron percnopterus, Aquila Chrysaetos, Columba livia*, *Streptopelia orientalis*, *Spilopelia chinensis*, *Psittacula eupatria*, *Otus sunia, Upupa epops*, *Delichon dasypus*, *Hirundo rustica, Trochalopteron lineatum, Motacilla cinerea*, *Motacilla alba*, *Motacilla citreola, and Anas platyrhynchos domesticus*) have a zero similarity index**.** Moreover, out of 14 mammalian species, 14 species, i.e., *Panthera pardus*, *Moschus chrysogaster*, *Ursus thibetanus, Hystrix indica, Macaca mulatta, Ovis aries*, *Canis aureus, Vulpes Vulpes, Petaurista petaurista, Bos Taurus, Oryctolagus cuniculus,* and *Bubalus bubalis,* have a zero similarity index**.** Additionally, out of five herpetofauna species, only two species, i.e., *Duttaphrynus melanostictus* and *Eublepharis macularius,* have a zero similarity index**.** Furthermore, it is noted that all species (i.e., *Paraconophyma* spp., *Meranoplus bicolor, Actias selene*, *Luciolasubstriata, Apis mellifera*, *Androctonus* spp., and *Libythea lepita*) of arthropods have a zero similarity index. Single species of earthworm also has a zero similarity index. Flesh, fat, bone, whole body, milk, skin, egg, head, feather, bile, blood, and honey were utilized as body parts. *Lerwa lerwa, Tragopan melanocephalus, Coturnix japonica*, *Alectoris chukar, Francolinus pondicerianus, Pucrasia macrolopha, Lophophorus impejanus*, *Lophura leucomelanos, Bubulcus ibis*, *Neophron percnopterus, Psittacula eupatria*, *Otussunia*, *Parus major*, *Delichon dasypus*, *Hirundo rustica, Motacilla cinerea, Motacilla alba*, *Motacilla citreola, Petaurista petaurista, Paraconophyma* spp., *Meranoplus bicolor, Actiasselene, Luciola substriata, Androctonus* spp., and *Libythea lepita* were noted for the first time from Himalayan region, AJ&K. This study gives information that could be useful in the conservation of animal biodiversity in Azad Jammu and Kashmir’s Himalayan region. For wild animal-based new pharmaceuticals, the screening of medicinal-active compounds and either “*in vivo*” or “*in vitro*” examination of biological activities of fauna with maximal “FC,” “UV,” “RI,” and “SI” could be relevant.

## Conclusion

To the best of our knowledge, ethnomedicinal uses of the diverse fauna of the Himalayan regions of Azad Jammu and Kashmir have rarely been reported before. Our findings revealed that local inhabitants have strong associations with animal species in their surrounding environment and use them in their primary health-care system to treat various diseases. In addition, medicinal uses of more than 60% of the species were reported for the first time from this area. Animal species with high medicinal values should be further explored for bioactive compounds and *in vitro/in vivo* activates to introduce novel animal-based health-care products. *Bos taurus* was documented as the most often consumed with FC = 29, while *Coturnix Coturnix* and *Columba livia* were documented to be highly versatile in their utilization (RI = 3.45) in the Himalayan region of Azad Jammu and Kashmir.

## Data Availability

The original contributions presented in the study are included in the article/Supplementary Material; further inquiries can be directed to the corresponding authors.
