# Supplementary material for: Traditional Uses of Animals in the Himalayan Region of Azad Jammu and Kashmir
Source: Front Pharmacol. 2022 Jun 29;13:807831. doi: 10.3389/fphar.2022.807831 (PMC9277021; doi:10.3389/fphar.2022.807831)
Supplement: Supplementary file 1 [file Table1.docx]

**Table 1 |** Ethnopharmacological uses among the people of district Haveli, Azad Jammu and Kashmir, Pakistan

| **Sr.** | **Scientific name, Common name, Local name** | **PU** | **MA** | **Treatments** | **Code** | **FC** | **UV** | **RI** | **SI** | **Reported use** | **References** |
| --- | --- | --- | --- | --- | --- | --- | --- | --- | --- | --- | --- |
| **Birds** | | | | | | | | | | | |
| 1 | *Lerwa lerwa* (Hodgson, 1833)  Snow Partridge, BarfaniTitri | F | O | **Fever** | SWF | 3 | 0.75 | 2.59 | 0 |  |  |
| 2 | *Tragopan melanocephalus* (Gray, 1829)  Western Tragopan, Rattgalla | F | O | **Lungs problems** | WTA | 1 | 0.25 | 0.86 | 0 |  |  |
|  |  |  |  | **Weakness** | WTP | 3 | 0.75 | 2.59 |  |  |  |
| 3 | *Coturnix coturnix* (Linnaeus, 1758)  Common Quail, Btair | F | O | **Bilious** | CQB | 4 | 1.00 | 3.45 | 0 | Body weakness, enhance memory, skin diseases, anemia, fever, sexual power | (Lohani, 2011b; Vijayakumar et al., 2015a, b; Altaf et al., 2017) |
|  |  |  |  | **Heart problems** | CQHD | 1 | 0.25 | 0.86 |  |  |  |
|  |  |  |  | **TB** | CQTB | 1 | 0.25 | 0.86 |  |  |  |
|  |  |  |  | **Joint pain** | CQJP | 4 | 1.00 | 3.45 |  |  |  |
|  |  |  |  | **Backbone pain** | CQBP | 4 | 1.00 | 3.45 |  |  |  |
|  |  |  |  | **Paralysis** | CQPL | 4 | 1.00 | 3.45 |  |  |  |
| 4 | *Coturnix japonica* Temminck & Schlegel, 1849  Rain Quail, Haum | F, B | O | **Regulate blood chemical** | RQIR | 1 | 0.50 | 1.72 | 0 |  |  |
| 5 | *Alectoris Chukar* (Gray, 1830)  Chukar Partridge, Chukor | F, B | O | **Weakness** | CQWN | 6 | 0.86 | 2.96 | 0 |  |  |
| 6 | *Francolinus francolinus*  (Linnaeus, 1766)  Black Francolin, Kala Teetar | F | O | **Joint pain** | BFOP | 2 | 0.67 | 2.30 | 0 | Bronchitis | (Arshad et al., 2014) |
|  |  |  |  | **Lungs problems** | BFBC | 2 | 0.67 | 2.30 |  |  |  |
| 7 | *Francolinu spondicerianus*  (Gmelin, 1789)  Grey Francolin, Bhoora Teetar | F | O | **Weakness** | GFWN | 3 | 0.75 | 2.59 | 0 |  |  |
| 8 | *Pucrasia macrolopha* (Lesson, 1829)  Koklas Peasant, Baiger | Ft | T | **Joints pain** | KPJP | 1 | 0.50 | 1.72 | 0 |  |  |
| 9 | *Lophophorus impejanus*  (Latham, 1790)  Himalayan Monal, Lainth | F | O | **Weakness** | HMWN | 8 | 0.89 | 3.07 | 0 |  |  |
| 10 | *Lophura leucomelanos* (Latham, 1790)  Kalij Pheasant, Bankukar | F | O | **Weakness** | KPWN | 6 | 0.86 | 2.96 | 0 |  |  |
|  |  |  |  | **Fever** | KPFV | 2 | 0.29 | 0.99 |  |  |  |
|  |  |  |  | **Memory** | KPNP | 2 | 0.29 | 0.99 |  |  |  |
| 11 | *Bubulcus ibis* (Linnaeus, 1758)  Cattle Egret, Raiyarro | F, Ft | O | **Memory** | CEMM | 1 | 0.50 | 1.72 | 0 |  |  |
|  |  |  |  | **Epilepsy** | CEEL | 1 | 0.50 | 1.72 |  |  |  |
| 12 | *Egretta garzetta* (Linnaeus, 1766)  Little Egret, Hanj | F, Ft | O | **Memory** | LEMM | 1 | 0.50 | 1.72 | 0 | Asthma, body strength, breathing trouble, immune enhancer | (Vijayakumar et al., 2015a, b) |
|  |  |  |  | **Epilepsy** | LEEL | 1 | 0.50 | 1.72 |  |  |  |
| 13 | *Neophron percnopterus* (Linnaeus, 1758)  White-rumped Vulture, Gidh | B | O | **Stomach problems** | WVSD | 3 | 0.75 | 2.59 | 0 |  |  |
|  |  |  |  | **Kidney problems** | WVKP | 3 | 0.75 | 2.59 |  |  |  |
|  |  |  |  | **Heart problems** | WVHA | 1 | 0.25 | 0.86 |  |  |  |
| 14 | *Aquila Chrysaetos* (Linnaeus, 1758)  Golden Eagle, Baz | Fr | T | **Wound healing** | GEWH | 4 | 0.80 | 2.76 | 0 | Abscess, anemia, epilepsy, infertility, Menorrhagia, Bronchitis, paralysis, puberty in young girls | (Benarjee et al., 2010; Alonso-Castro et al., 2011; Jacobo-Salcedoa et al., 2011Arshad et al., 2014; Paudyal and Singh, 2014; Vijayakumar et al., 2015a, b; Altaf et al., 2017) |
|  |  |  |  | **Regulate blood chemical** | GEBP | 1 | 0.20 | 0.69 |  |  |  |
| 15 | *Columba livia*  (Gmelin, 1789)  Common Pigeon, Kalbootar | F, E | O | **Parkinson's disease** | CPPD | 18 | 1.00 | 3.45 | 0 | Abscess, anemia, Bronchitis, epilepsy, infertility, Menorrhagia, paralysis, puberty in young girls, | (Benarjee et al., 2010; Alonso-Castro et al., 2011; Chakravorty et al., 2011; Bagde and Jain, 2013; Mootoosamy and Mahomoodally, 2014; Altaf et al., 2017) |
|  |  |  |  | **Ptosis** | CPPT | 6 | 0.33 | 1.15 |  |  |  |
|  |  |  |  | **Tongue problem** | CPTP | 6 | 0.33 | 1.15 |  |  |  |
| 16 | *Columba rupestris* Pallas, 1811  Hill Pigeon, Jangli kalbootar | F, B | O | Wound healing | HPWH | 18 | 1.00 | 3.45 | 1 | Wound healing | (Mughal et al., 2020) |
| 17 | *Streptopelia orientalis* (Latham, 1790)  Oriental Turtle Dove, Kogath | F | O | **Paralysis** | ODPL | 9 | 0.90 | 3.10 | 0 | Impotency, girls maturity | (Altaf et al., 2017) |
|  |  |  |  | **Enhance muscle power** | ODEP | 9 | 0.90 | 3.10 |  |  |  |
| 18 | *Spilopeliachinensis* (Scopoli, 1786)  Spotted Dove, Kamerri | F | O | **Paralysis** | SDPL | 9 | 0.75 | 2.59 | 0 | Impotency, girls maturity | (Altaf et al., 2017) |
|  |  |  |  | **Enhance muscle power** | SDEP | 9 | 0.75 | 2.59 |  |  |  |
| 19 | *Psittacula eupatria* (Linnaeus, 1766)  Alexander Parakeet, Totta | Ft, B | T | **Memory** | APEM | 2 | 0.67 | 2.30 |  |  |  |
| 20 | *Eudynamys scolopaceus* (Linnaeu, 1758)  Asian Koel, Koel | F | O | Spleen problem | AKSP | 1 | 0.50 | 1.72 | 1 | Spleen problem | (Mughal et al., 2020) |
| 21 | *Otus sunia* (Hodgson, 1836)  Oriental Scopus Owl, Ullo | F, B | T | **Whooping cough** | OWWC | 2 | 0.67 | 2.30 | 0 |  |  |
| 22 | *Upupa epops* (Linnaeus, 1758)  Common Hoopoe, Mahiya | F, Bl | O | **Stomach problems** | CHCD | 10 | 0.83 | 2.87 | 0 | Gall bladder stone, kidney problems | (Betlu, 2013; Altaf et al., 2017) |
|  |  |  |  | **Liver disease** | CHLD | 2 | 0.17 | 0.57 |  |  |  |
|  |  |  |  | **Bladder disease** | CHBD | 2 | 0.17 | 0.57 |  |  |  |
|  |  |  |  | **Eyesight** | CHEE | 2 | 0.17 | 0.57 |  |  |  |
| 23 | *Corvus splendens* (Vieillot, 1817)  House Crow, Kagh | Bd | T | Piles | HCPL | 1 | 0.50 | 1.72 | 1 | Piles | (Mughal et al., 2020) |
| 24 | *Parus major* (Linnaeus, 1758)  Great Tit  Tit | F, Ft | O, T | **Male impotency** | GTMP | 4 | 0.80 | 2.76 | 0 |  |  |
|  |  |  |  | **Skin Problem** | GTSP | 4 | 0.80 | 2.76 |  |  |  |
| 25 | *Delichon dasypus*  (Bonaparte, 1850)  Asian House Martin, Bulbl | S | O | **Male impotency** | AMMP | 1 | 0.50 | 1.72 | 0 |  |  |
| 26 | *Hirundo rustica*  (Linnaeus, 1758)  Barn Swallow, Ababil | S | O | **Male impotency** | BSMP | 1 | 0.50 | 1.72 | 0 |  |  |
| 27 | *Trochalopteron lineatum* (Vigors, 1831)  Streaked Laughingthrush, Sorri | F, B | O | **Weakness** | SLBS | 5 | 0.71 | 2.46 | 0 | Paralysis, flue and fever | (Hakeem et al., 2017) |
| 28 | *Acridotheres tristis* (Linnaeus, 1766)  Common Myna, Gotaari | F | O | Whooping cough | CMWC | 4 | 0.80 | 2.76 | 1 | Whooping cough, weakness | (Altaf et al., 2017) |
|  |  |  |  | Weakness | CMWN | 4 | 0.67 | 2.30 |  |  |  |
| 29 | *Passer domesticus* (Linnaeus, 1758)  House Sparrow, Chirri | F, Br | O | Male impotency | HSMI | 12 | 1.00 | 3.45 | 0.2 | Allergy, aphrodisiac, chickenpox, constipation, delay dentition, fever, gas trouble, increase sexual desire, paralysis, impotency, weakness, wound healing | (Lohani, 2011b; Arshad et al., 2014; Aloufi and Eid, 2016; Mughal et al., 2020) |
|  |  |  |  | Paralysis | HSPL | 12 | 1.00 | 3.45 |  |  |  |
|  |  |  |  | Measles | HSML | 1 | 0.08 | 0.29 |  |  |  |
|  |  |  |  | Stomach problems | HSCP | 1 | 0.08 | 0.29 |  |  |  |
| 30 | *Passer cinnamomeus* (Temminck, 1836)  Russet Sparrow,Junglichirri | F | O | Paralysis | RSPL | 12 | 1.00 | 3.45 | 0.25 | Paralysis, joints problem | (Rauf et al., 2017) |
|  |  |  |  | Male impotency | RSMI | 12 | 1.00 | 3.45 |  |  |  |
|  |  |  |  | Liver diseases | RSLD | 2 | 0.17 | 0.57 |  |  |  |
| 31 | *Motacilla cinerea* (Tunstall, 1771)  Grey wagtail, Chirri | F | O | **Kidney problems** | GWKS | 1 | 0.33 | 1.15 | 0 |  |  |
| 32 | *Motacilla alba* (Linnaeus, 1758)  White wagtail, Chirri | F | O | **Kidney problems** | WWKS | 1 | 0.25 | 0.86 | 0 |  |  |
| 33 | *Motacilla citreola* (Pallas, 1776)  Citrine wagtail, Chirri | F | O | **Kidney problems** | CWKS | 1 | 0.33 | 1.15 | 0 |  |  |
| 34 | *Anas platyrhynchos domesticus* (Linnaeus, 1758)  Duck, Batakh | F, Ft, E | O | **Kidney Problems** | DUKP | 19 | 1.00 | 3.45 | 0 | Body strength, scarlet fever, weakness, erectile dysfunction, | (Alves et al., 2012; Vijayakumar et al., 2015a, b) |
|  |  |  |  | **Heart problems** | DUHP | 19 | 1.00 | 3.45 |  |  |  |
|  |  |  |  | **BP** | DUBP | 8 | 0.42 | 1.45 |  |  |  |
|  |  |  |  | **Male impotency** | DUMI | 10 | 0.53 | 1.81 |  |  |  |
|  |  |  |  | **Piles** | DUPL | 3 | 0.16 | 0.54 |  |  |  |
|  |  |  |  | **Blindness** | DUBN | 2 | 0.11 | 0.36 |  |  |  |
|  |  |  |  | **Eyesight** | DUES | 1 | 0.05 | 0.18 |  |  |  |
| 35 | *Gallus gallus domesticus* (Linnaeus, 1758)  Hen, Murghi | F, E | O | Kidney Problems | HNKP | 24 | 1.00 | 3.45 | 0.067 | Asthma, bronchitis, bronchitis, burns, burst furuncles, diabetes, evil eye, eye-problems, flu, furuncle, indigestion, sinusitis, nervous problems, rheumatism, night blindness, nourishing food, optic infection, shortness of breath, sore throat, sprains, strains, stuffy nose, weak bones, weakness | (Oliveira et al., 2010; Lohani, 2011b; Alves, 2012; Barros et al., 2012; Haileselasie, 2012; Kim and Song, 2013; Bagde and Jain, 2015) |
|  |  |  |  | Hearts problems | HNHP | 16 | 0.67 | 2.30 |  |  |  |
|  |  |  |  | Weakness | HNSM | 19 | 0.79 | 2.73 |  |  |  |
|  |  |  |  | Memory | HNEM | 19 | 0.79 | 2.73 |  |  |  |
|  |  |  |  | Eyesight | HNES | 19 | 0.79 | 2.73 |  |  |  |
|  |  |  |  | Male impotency | HNMI | 24 | 1.00 | 3.45 |  |  |  |
|  |  |  |  | Diabetes | HNDB | 7 | 0.29 | 1.01 |  |  |  |
|  |  |  |  | Stomach problems | HNDR | 2 | 0.08 | 0.29 |  |  |  |
|  |  |  |  | BP | HNBP | 24 | 1.00 | 3.45 |  |  |  |
| Mammals | | | | | | | | | | | |
| 36 | *Panthera pardus* (Linnaeus, 1758)  Common Leopard, Cheetah | S, F | T | **Measles** | PPML | 5 | 0.33 | 1.15 | 0 | Rheumatism. | (Nijman and Shepherd, 2017) |
|  |  |  |  | **Chicken pox** | PPCP | 5 | 1.00 | 3.45 |  |  |  |
|  |  |  |  | **Male impotency** | PPMI | 5 | 1.00 | 3.45 |  |  |  |
| 37 | *Moschus chrysogaster* (Hodgson, 1839)  Alpine musk deer, Roansa | F | O | **Paralysis** | MCPL | 6 | 0.46 | 1.59 | 0 |  |  |
| 38 | *Ursus thibetanus* (G. Baron Cuvier,1823)  Asiatic Black Bear*,* Rech | Ft | T | **Joint pain** | UTJP | 4 | 0.44 | 1.53 | 0 | Treatment of children mouth disease and injuries, heal diarrhea, remove and heal degenerated tissues. | (Nijman and Shepherd, 2017) |
|  |  |  |  | **Male impotency** | UTMI | 5 | 0.56 | 1.92 |  |  |  |
| 39 | *Hystrix indica* (Kerr, 179)  Indian crested porcupine  Saig | F, Ft | T | **Joint pain** | IPJP | 2 | 0.40 | 1.38 | 0 | Asthma, boiled, colic, easy delivery of a child, foot mouth disease, muscle fatigue, pre menstrual pain, rheumatic pain, Skin infection, stomach-ache, weakness, | (Lohani, 2010; 2011b; Mishra et al., 2011; Bagde and Jain, 2013; Betlu, 2013; Galave et al., 2013; Aloufi and Eid, 2016; Altaf et al., 2017; Borah and Prasad, 2017; Altaf et al., 2018) |
| 40 | *Macaca mulatta* (Zimmermann, 1780)  Rhesus Macaque, Bandar | F | T | **Wounds** | RMW | 1 | 0.33 | 1.15 | 0 |  |  |
| 41 | *Canis aureus* (Linnaeus, 1758)  Asiatic jackal, Gedar | Ft | O | **Skin problems** | AJSD | 2 | 0.40 | 1.38 | 0 | Rheumatic pain and body ache | (Benarjee et al., 2010; Chakravorty et al., 2011; Lohani, 2011b; a; Betlu, 2013; Chinlampianga et al., 2013; Paudyal and Singh, 2014; Altaf et al., 2018) |
| 42 | *Vulpes vulpes* (Linnaeus, 1758)  Red fox, Phand | F | O | **Male impotency** | RFMI | 1 | 0.25 | 0.86 | 0 |  |  |
| 43 | *Petaurista petaurista* (Pallas, 1766)  Giant redHimalayan squirrel, Keese | F | O | **Diabetes** | HSDB | 2 | 0.33 | 1.15 | 0 |  |  |
| 44 | *Bos taurus* (Linnaeus, 1758)  Cow, Gay | F, M | O | **Enhance protein** | COEP | 29 | 1.00 | 3.45 | 0 | Asthma, bone, fever, diarrhea, eye infection, gastritis, memory loss, paralysis, pesticide, stomach ache, tuberculosis | (Dixit et al., 2010; Lohani, 2010; 2011a; b; Mishra et al., 2011; Bagde and Jain, 2015) |
|  |  |  |  | **Weakness** | COPH | 29 | 1.00 | 3.45 |  |  |  |
|  |  |  |  | **Boil** | COBL | 12 | 0.41 | 1.43 |  |  |  |
| 45 | *Oryctolagus cuniculus* (Lilljeborg, 1873)  Rabit, Khargosh | F | O | **Ptosis** | RBPT | 5 | 0.83 | 2.87 | 0 | Stomach pain, bronchial problems | (Alonso-Castro et al., 2011) |
|  |  |  |  | **Epilepsy** | RBEL | 5 | 0.83 | 2.87 |  |  |  |
|  |  |  |  | **Whooping cough** | RBWC | 5 | 0.83 | 2.87 |  |  |  |
|  |  |  |  | **Boil** | RBBL | 5 | 0.83 | 2.87 |  |  |  |
| 46 | *Bubalus bubalis* (Linnaeus, 1758)  Buffalo, Maj | F, M | O | **Enhance protein** | BFEP | 21 | 0.83 | 2.87 | 0 | Ascites, jaundice, osteoporosis, Pain, wound, rheumatic pain, thrombosis, weakness | (Alves and Rosa, 2007; Alves et al., 2007; Benarjee et al., 2010; Dixit et al., 2010; Vijayakumar et al., 2015a, b) |
|  |  |  |  | **Fever** | BFFV | 21 | 0.91 | 3.15 |  |  |  |
| 47 | *Camelus dromedaries* (Linnaeus, 1758)  Camel, Aunth | M | O | Piles | CMPL | 8 | 0.80 | 2.76 | 0.17 | Acidity, hepatitis B and C | (Jaroli et al., 2010; Arshad et al., 2014) |
|  |  |  |  | Hepatitis B and C | CMCB | 8 | 0.80 | 2.76 |  |  |  |
|  |  |  |  | Male impotency | CMMI | 8 | 0.80 | 2.76 |  |  |  |
|  |  |  |  | Bladder problems | CMBP | 8 | 0.80 | 2.76 |  |  |  |
|  |  |  |  | Boil | CMB | 8 | 0.80 | 2.76 |  |  |  |
| 48 | *Capra aegagrus hircus* (Linnaeus, 1758)  Goat, Bakri | F, M | O | BP | GTBP | 6 | 0.75 | 2.59 | 0.056 | Anemia, asthma, tuberculosis, blindness, bronchitis, jaundice, diarrhea, dysentery, eye tonic, Fever, menstrual, disorder, tonsillitis, toothache | (Jaroli et al., 2010; Arshad et al., 2014; Chellappandian et al., 2014; Bagde and Jain, 2015; Vijayakumar et al., 2015a) |
|  |  |  |  | Regulate blood chemical | GTRB | 6 | 0.75 | 2.59 |  |  |  |
|  |  |  |  | Liver diseases | GTLD | 6 | 0.75 | 2.59 |  |  |  |
|  |  |  |  | Blindness | GTBN | 6 | 0.75 | 2.59 |  |  |  |
|  |  |  |  | Kidney diseases | GTLD | 6 | 0.75 | 2.59 |  |  |  |
|  |  |  |  | Boil | GTBL | 6 | 0.75 | 2.59 |  |  |  |
| 49 | *Ovis aries* (Linnaeus, 1758)  Sheep, Bhairh | F | O | **BP** | SPBP | 9 | 0.82 | 2.82 | 0 | Weakness, edema, flu, fractures, joint pain, muscular pain, skin burn, crack, sterility, swellings | (Alves et al., 2009; Oliveira et al., 2010; Benítez, 2011; Lohani, 2011b; Alves, 2012; Melo et al., 2014) |
|  |  |  |  | **Regulate blood chemical** | SPBP | 9 | 0.82 | 2.82 |  |  |  |
|  |  |  |  | **Liver diseases** | SPBP | 9 | 0.82 | 2.82 |  |  |  |
| **Herptiles** | | | | | | | | | | | |
| 50 | *Duttaphrynus melanostictus* (Schneider, 1799)  Hazaragauk, Dad | Ft | T | **Antibacterial** | HGAB | 2 | 0.40 | 1.38 | 0 |  |  |
|  |  |  |  | **Antifungal** | HGAF | 2 | 0.40 | 1.38 |  |  |  |
| 51 | *Laudakia agrorensis* (Stoliczka, 1872)  Agror agama, Sandaila | Ft | T | Joint pain | LAJP | 3 | 0.33 | 1.15 | 0.2 | Arthritis, burn, cough, fever, jaundice, joint pain, malaria, sexual stimulant, skin disease | (Padmanabhan and Sujana, 2008; Benarjee et al., 2010; Chakravorty et al., 2011; Lalmuanpuii et al., 2013; Borah and Prasad, 2017) |
|  |  |  |  | Backbone pain | LABB | 3 | 0.33 | 1.15 |  |  |  |
|  |  |  |  | Male impotency | LAMI | 3 | 0.33 | 1.15 |  |  |  |
| 52 | *Naja oxiana* Eichwald, 1837  Brown cobra, Chajla sup | Ft, S | T | Joint pain | BNJP | 4 | 0.40 | 1.38 | 0.34 | Visual illnesses | (Aloufi and Eid, 2016; Ajagun et al., 2017) |
|  |  |  |  | Piles | BNPL | 4 | 0.40 | 1.38 |  |  |  |
|  |  |  |  | Eyesight | BNES | 2 | 0.20 | 0.69 |  |  |  |
| 53 | *Calotes versicolo r*(Daudin, 1802)  Oriental Garden Lizard, Girgit | Ft | T | Joint pain | GLJP | 1 | 0.25 | 0.86 | 0.2 | Visual, joint pain, blood, foot and toes injuries | (Dixit et al., 2010; Vijayakumar et al., 2015b; Aloufi and Eid, 2016; Altaf, 2020) |
| 54 | *Eublepharis macularius* (Börner, 1981)  Leopard gecko, PanjgiraSandaila | Ft | T | **Cancer** | LGCC | 2 | 0.40 | 1.38 | 0 |  |  |
| **Arthropods** | | | | | | | | | | | |
| 55 | *Paraconophyma* spp. (Uvarov, 1921)  Locust, Tidi | WB | T | **Lungs problems** | LCLP | 2 | 0.33 | 1.15 | 0 |  |  |
|  |  |  |  | **Antibacterial** | LCAB | 2 | 0.33 | 1.15 |  |  |  |
|  |  |  |  | **Antifungal** | LCAF | 2 | 0.33 | 1.15 |  |  |  |
| 56 | *Meranoplus bicolor* (Guérin-Méneville, 1844)  Ants, Chownti | H | T | **Deafness** | BADN | 3 | 0.43 | 1.48 | 0 |  |  |
| 57 | *Actias selene* (Hübner, 1807)  Moth, Titli | H | T | **Antibacterial** | SADN | 3 | 0.38 | 1.29 | 0 |  |  |
| 58 | *Luciolasubstriata* Gorham, (1880)  Firefly, Jugno | WB | T | **Ear problem** | FFEP | 1 | 0.20 | 0.69 | 0 |  |  |
| 59 | *Apis mellifera* (Linnaeus, 1758)  Honey bee, Shahad di makhi | HN | O | **Diabetes** | HBDB | 8 | 0.89 | 3.07 | 0 | Aging, arthritis, bronchitis, burn, cataract, cold, constipation, cough, dark spots, fever, flu, sexual impotence, shortness of breath, skin lightening, sore throat, tuberculosis | (Alves et al., 2010; Oliveira et al., 2010; Yirga et al., 2011; Alves, 2012; Haileselasie, 2012; Martínez, 2013; Mootoosamy and Mahomoodally, 2014; Vats and Thomas, 2015; Aloufi and Eid, 2016; Umair and Yaqoob, 2018; Altaf and Umair, 2020) |
|  |  |  |  | **Stomach problems** | HBUL | 8 | 0.89 | 3.07 |  |  |  |
|  |  |  |  | **Eyesight** | HBES | 8 | 0.89 | 3.07 |  |  |  |
| 60 | *Androctonus* spp. (Ehrenberg, 1828)  Scorpion, Bicho | WB | T | **Cancer** | SPCC | 2 | 0.40 | 1.38 | 0 |  |  |
| 61 | *Libythea lepita* [Moore, 1857](https://en.wikipedia.org/wiki/Frederic_Moore)  Common beak, Titli | WB | T | **Antibacterial** | CBAB | 2 | 0.33 | 1.15 | 0 |  |  |
|  |  |  |  | **Antifungal** | CBAF | 2 | 0.33 | 1.15 |  |  |  |
| **Earthworm** | | | | | | | | | | | |
| 62 | *Pheretima hawayana* Rosa, 1891  Earthworm, Gandoya | WB | T | **Backbone pain** | EWBB | 4 | 0.40 | 1.38 | 0 | Wound, impotence | (Mootoosamy and Mahomoodally, 2014; Vats and Thomas, 2015) |

**Note:** F (flesh), Ft (fat), Fr (feather), E (egg), B (bones), M (milk), WB (whole body), H (head), HN (honey), Bl (bile), Bd (blood), S (skin), Br (brain), O (oral), T (topical). ***Bold:** Medicinal uses which are reported for very first time in this study.
